# Discovery of novel transcripts and gametophytic functions via RNA-seq analysis of maize gametophytic transcriptomes

**DOI:** 10.1186/s13059-014-0414-2

**Published:** 2014-07-31

**Authors:** Antony M Chettoor, Scott A Givan, Rex A Cole, Clayton T Coker, Erica Unger-Wallace, Zuzana Vejlupkova, Erik Vollbrecht, John E Fowler, Matthew MS Evans

**Affiliations:** Department of Plant Biology, Carnegie Institution for Science, Stanford, CA 94305 USA; Informatics Research Core Facility, University of Missouri, Columbia, MO 65211 USA; Department of Botany and Plant Pathology, Oregon State University, Corvallis, OR 97331 USA; Department of Genetics, Development and Cell Biology, Iowa State University, Ames, IA 50011 USA

## Abstract

**Background:**

Plant gametophytes play central roles in sexual reproduction. A hallmark of the plant life cycle is that gene expression is required in the haploid gametophytes. Consequently, many mutant phenotypes are expressed in this phase.

**Results:**

We perform a quantitative RNA-seq analysis of embryo sacs, comparator ovules with the embryo sacs removed, mature pollen, and seedlings to assist the identification of gametophyte functions in maize. Expression levels were determined for annotated genes in both gametophytes, and novel transcripts were identified from *de novo* assembly of RNA-seq reads. Transposon-related transcripts are present in high levels in both gametophytes, suggesting a connection between gamete production and transposon expression in maize not previously identified in any female gametophytes. Two classes of small signaling proteins and several transcription factor gene families are enriched in gametophyte transcriptomes. Expression patterns of maize genes with duplicates in subgenome 1 and subgenome 2 indicate that pollen-expressed genes in subgenome 2 are retained at a higher rate than subgenome 2 genes with other expression patterns. Analysis of available insertion mutant collections shows a statistically significant deficit in insertions in gametophyte-expressed genes.

**Conclusions:**

This analysis, the first RNA-seq study to compare both gametophytes in a monocot, identifies maize gametophyte functions, gametophyte expression of transposon-related sequences, and unannotated, novel transcripts. Reduced recovery of mutations in gametophyte-expressed genes is supporting evidence for their function in the gametophytes. Expression patterns of extant, duplicated maize genes reveals that selective pressures based on male gametophytic function have likely had a disproportionate effect on plant genomes.

**Electronic supplementary material:**

The online version of this article (doi:10.1186/s13059-014-0414-2) contains supplementary material, which is available to authorized users.

## Background

The plant life cycle has genetically active diploid and haploid phases, called the sporophyte and gametophyte, respectively [1]. In angiosperms the gametophytes are highly reduced, are dependent on the parent sporophyte, and develop embedded within the diploid sporophyte tissues, with a three-celled male gametophyte and a female gametophyte consisting of as few as seven cells.

To produce the female gametophyte, or embryo sac, after meiosis, one spore undergoes three rounds of synchronous divisions to produce an eight-nucleate syncytium with micropylar and chalazal clusters of four nuclei each [2]. Cellularization then produces seven cells: two synergids, the egg cell, the bi-nucleate central cell, and three antipodal cells [3]. In maize, the antipodal cells continue to divide during embryo sac maturation, reaching a final number of 20 to 100 cells. The male gametophyte, or pollen grain, has an even more reduced phase of growth. Each microspore first undergoes an asymmetric cell division to produce the vegetative cell and the generative cell. The generative cell then divides once to produce the two sperm cells, which are carried within the vegetative cell. In addition to expressing functions required for pollen grain development, the vegetative cell must also generate the tip-growing pollen tube that navigates through the pistil tissues to reach the embryo sac and deliver the sperm cells [4].

Mutations in genes required in the gametophytes result in characteristic fertility phenotypes and modes of transmission that have formed the basis of many mutant screens [5-9]. When heterozygous, mutations affecting the embryo sac are expected to have reduced fertility and seed set, because half of the ovules contain mutant embryo sacs and so often fail to produce seed. Mutations affecting the male gametophyte do not cause reduced seed set, because both wild-type and mutant pollen from heterozygotes enter the pistil. However, for mutations affecting male and/or female gametophytes, the mutant allele (and the alleles of loci linked to it) is found at a reduced frequency in progeny when the defective gamete is involved (that is, male gametophyte mutants are recovered poorly when heterozygotes are crossed as males). This characteristic reduced transmission also prevents, or makes very difficult, the generation of mutant homozygotes. Note that genetic redundancy can facilitate the recovery of mutations in genes active in the gametophytes but also can complicate recognizing them as such, given generally weaker phenotypes. Maize, as an ancient allotetraploid constituted by two progenitor genomes (subgenomes 1 and 2), has a mix of genes present as either duplicated pairs (homeologs), or as singletons, due to gene loss [10]. Notably, subgenome 2 is characterized by lower levels of gene expression and higher rates of gene loss than subgenome 1 [11].

Because of the poor recovery of gametophyte-lethal mutants, additional strategies (for example, transcriptome profiling) have been utilized to identify gametophyte active genes in several species. Microarrays were used first to assess the transcriptomes of pollen [12-15] and embryo sacs (by comparing ovules with and without embryo sacs) [16-21] in *Arabidopsis*. These studies identified up to approximately 14,000 genes as expressed at some point in pollen development [13], with approximately 6,500 to 7,200 expressed at the mature pollen stage [13,15] and 1,200 embryo sac-expressed genes. The identification of more expressed genes in pollen is likely due to the ease of isolating large populations of relatively pure material. Sperm cell purification and assessment of growing pollen tubes have extended these studies to provide additional details of male gametophytic transcriptomes [22-25].

Enrichment of embryo sac cells (for example, by cell wall digestion and dissection [26] or laser capture microdissection [27]) facilitated the identification of additional genes expressed in the embryo sac. Isolation of gametophyte cells for expressed sequence tag sequencing or microarray hybridization in maize, rice, and wheat [26,28-31] identified greater complexity for the egg transcriptome than that of sperm, and a preponderance of unknown, hypothetical, and novel proteins encoded by these transcripts. Three of the cell types in the mature *Arabidopsis* embryo sac (the egg cell, the central cell and the synergids; but not the antipodals) were analyzed by microarray, with 8,850/20,777 of the genes on the ATH1 chip identified as expressed [27], a number comparable to mature pollen. RNA-seq analysis removes some of the limitations associated with sequencing individual cDNA clones or microarray technology (for example, not all of the genes are present on the microarray), revealing both the expression of a higher fraction of known transcripts in the gametophytes and the existence of new genes and transcript isoforms in mature pollen and the central cell of the female gametophyte [32-34]. RNA-seq has also identified gene families enriched in the central cell that were missed in microarray studies [35].

These studies have revealed a few broad themes. The pollen transcriptome is the most distinctive, although all gametophytic transcriptomes have some similar features to one another. Of sporophytic transcriptomes, the early embryo (heart and globular stages) is most similar to the gametophytes [15,24,27]. Some parallels for plant egg and sperm cell transcriptomes with animal gamete transcriptomes have also been detected, particularly with regards to the epigenetic regulation of gene function through small RNA pathways [27]. Within the embryo sac, the egg and central cell transcriptome are more similar to one another than to the synergids. Small signaling peptides of the DEFENSIN/LURE (DEFL) family are overrepresented in the female gametophyte (particularly the central cell), although only a subset of these was assayed in the whole embryo sac. Pollen grain transcriptomes are enriched for Gene Ontology (GO) terms related to signaling, vesicle trafficking, cell wall functions, and cytoskeletal functions thought to be important for tip growth [15,25]. Finally, putative connections between epigenetic regulation, small RNA pathways, and reactivation and silencing of transposable elements (TEs) have been observed in gametophyte transcriptomes [23,27,36]. Preliminary RNA-seq analysis is available for maize mature pollen [37], which, as is the case in *Arabidopsis*, is very different from sporophytic tissues. Use of *de novo* transcript assembly of RNA-seq reads in maize has also been used to study long non-coding RNAs in reproductive and vegetative tissues [38]. Reproductive tissues, including male and female gametophytes, express more long noncoding RNA (lncRNA) loci than vegetative tissues.

Here the first detailed, replicated, RNA-seq-based analysis of both male and female gametophytic transcriptomes of maize (or any monocot) is used to identify genome features with differential expression between the gametophytes and sporophytic tissues, including protein-coding gene families, duplicated genes, and previously unannotated genes. These studies identify small signaling peptides and several transcription factor (TF) gene families as being overrepresented in gametophyte transcriptomes. The first genome-wide comparison of gene expression patterns on duplicate gene retention also reveals an effect of pollen gene function on genome evolution. This study also provides the first evidence for transposon expression in the male and female gametophytes of a plant with a large, complex genome containing many active transposon classes.

## Results

### Production of RNA-seq and mapping of reads to the B73 reference genome

To define the transcriptomes of mature maize male and female gametophytes, RNA-seq was performed on four tissue types: nine-day old, above-ground seedling (S); mature pollen (MP); embryo-sac-enriched samples with some remaining nucellar cells (ES); and ovules with embryo sacs removed (Ov). We generated between approximately 54 million to approximately 195 million mappable Illumina reads per B73 sample. The ES samples, which had the lowest amount of starting material and required additional amplification before sequencing, had the lowest percentage of reads that could be mapped back to the reference genome, ranging from 54% to 62% of the total reads per replicate. Before mapping reads to the maize genome, reads were compared to the available maize repeat database to remove reads with a high confidence match to maize repetitive elements [39]. Remaining reads were mapped to the maize genome sequence in two ways: (1) to the existing gene models to determine expression levels for annotated genes; and (2) to the reference genome sequence independently of gene models to build empirical transcripts to aid the identification of novel genes. There are three gene sets for the maize B73 RefGen_v2: the filtered gene set (FGS), composed of high-confidence gene models; the rejected gene set (RGS), composed of lower-confidence gene models that include likely pseudogenes and transposons; and the working gene set (WGS), which encompasses both FGS and RGS. To ensure that unknown gametophytic transcripts were not missed in this analysis, B73 RNA-seq reads were mapped to both the WGS (Additional file 1) and FGS (Additional file 2) gene models (summarized in Table 1).

**Table 1:** **RNA-seq reads mapped backed to the maize genome (5a.59)**

	**B73 seedling**	**B73 mature pollen**	**B73 embryo sac**	**B73 ovule without embryo sac**	**W23 embryo sac**	**W23 ovule without embryo sac**
Percentage of reads mapped back to nuclear genome	82%	85%	61%	81%	ND	ND
Total mapped reads	91,076,832	123,536,281	50,435,150	140,282,423	ND	ND
Reads mapped to FGS low-copy exons	46,765,091	100,723,578	34,727,288	117,425,717	17,569,670	16,837,249
Reads mapped to RGS Low-copy exons	2,099,557	1,822,553	1,602,788	3,873,497		
Intron	3,214,112	367,752	1,110,429	2,277,426	ND	ND
Intergenic	1,522,581	2,732,794	5,583,100	4,199,131	ND	ND
Ribosomal RNA genes	21,039,136	4,306,489	729,102	4,224,584	ND	ND
Transposons and other repeats	8,256,280	12,504,295	3,590,804	3,629,696	ND	ND
Mitochondrial genes	3,442,450	49,613	1,399,727	2,450,967	ND	ND
Chloroplast genes	4,176,008	771	988,470	1,592,112	ND	ND
Genes in FGS with average expression >0.1 FPKM (39,635 total)	27,564	14,591	27,530	25,971	20,857	20,539
Genes in RGS with average expression >0.1 FPKM (69,689 total)	8,165	4,335	17,751	11,933	ND	ND
Percentage of FGS expressed genes (>0.1 FPKM) in only one replicate	9%	18%	16%	11%	19%	9%
Percentage of FGS expressed genes (>0.1 FPKM) in two replicates	8%	13%	17%	8%	15%	14%
Percentage of FGS expressed genes (>0.1 FPKM) in all three replicates	83%	69%	67%	81%	66%	77%

FPKM, fragments per kilobase per million reads; ND, not determined.

**Figure 1:**
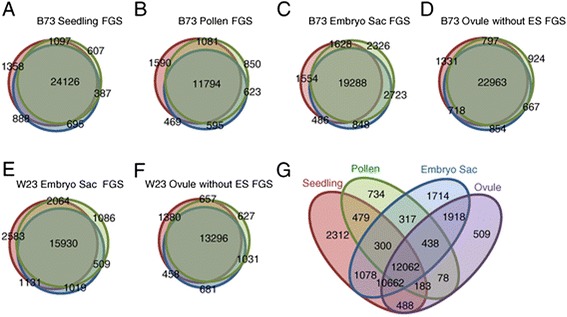
**Similarity between replicates.** The lists of genes with expression above 0.1 FPKM for each sample was compared between biological replicates. Overlaps between replicates within each tissue type are shown. The number of genes with an expression of at least 0.1 FPKM within each set or overlap between sets is indicated. The samples with the least overlap are the B73 embryo sacs. **(A)** B73 seedlings; **(B)** B73 mature pollen; **(C)** B73 embryo sac enriched; **(D)** B73 ovules without embryo sacs; **(E)** W23 embryo sac enriched; **(F)** W23 ovules without embryo sacs. **(G)** Overlap between lists of FGS genes with an average expression above 0.1 FPKM for each tissue type. FGS, filtered gene set.

The variability of samples can be seen in the percentages of gene models expressed above an arbitrary threshold (0.1 fragments per kilobase per million reads (FPKM)) that are shared between replicates (Table 1 and Figure 1). ES was the most variable. The percentage of genes shared across all three ES samples is lower than in the other tissues (67% versus 69 to 83%). Because of the variability of the ES samples, an additional set of ES and comparator Ov samples were analyzed to improve identification of genes enriched in the embryo sac. RNA-seq was performed on a set of samples from a different inbred line, W23, using the ABI SOLiD platform. These reads were mapped against the FGS genes, and FPKM values calculated for each gene (Additional file 2). The W23 ES samples had similar variability between replicates as the B73 ES (66% of genes above 0.1 FPKM are shared in all three). For those FGS genes with an average signal above threshold in the W23 samples, there was a strong concordance with the same characteristic in B73 (94% for ES, 93% for Ov), arguing that the samples from the two inbred lines are indeed comparable. All subsequent analysis of FGS gene expression values used a modified average of the W23 and B73 ES and Ov samples derived as described in the Materials and methods.

**Figure 2:**
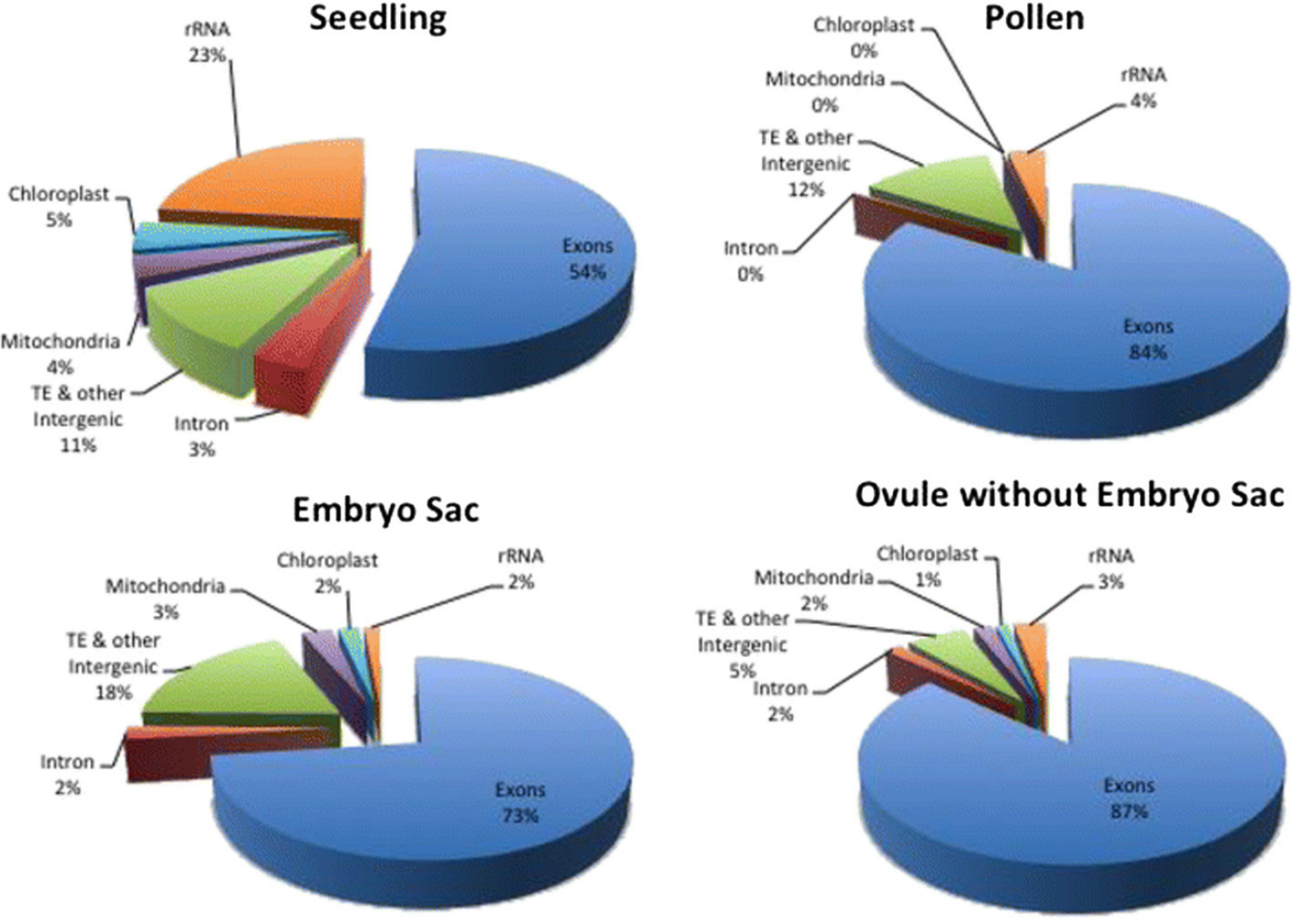
**Distribution of RNA-seq reads to different genomic features in maize.** The frequency of the reads mapping to transposable elements and other intergenic sequences (TE & other intergenic), ribosomal RNA genes (rRNA), other nuclear annotated gene model exons (exons), annotated gene introns, mitochondrial genes, and chloroplast genes. Dramatic differences were seen, with rRNA reads most abundant in the seedling tissue, TE & other intergenic transcripts lowest in the Ov, chloroplast and mitochondrial transcripts lowest in MP, and TE & other intergenic transcripts most abundant in ES.

Several trends involving genomic regions were revealed when reads were mapped to the whole genome (Table 1 and Figure 2). For example, the reads mapping to ribosomal sequences and the chloroplast genome were highest in seedling, whereas MP was nearly devoid of reads from both the mitochondrial and chloroplast genomes. Reads classified as intronic were also notably less frequent in MP (0.3%) than in the other tissues tested (2% to 3%). This is in contrast to *Arabidopsis* pollen, which has a high frequency of intron reads [33]. One possible explanation for the low representation of introns in MP is that, in contrast to the other sample types, mature pollen is in a somewhat quiescent state prior to contact with female tissue. Thus, this observation is consistent with the view that the vast majority of mRNAs in MP are fully mature (that is, completely spliced), and stored for rapid translation upon pollen tube germination [40].

Non-exonic reads (intron, TE and other intergenic) were overrepresented in ES samples relative to the other transcriptomes (approximately 50% more frequent than in seedling and MP and three-fold more frequent than in surrounding Ov), raising the possibility that ES-specific genes have been systematically missed in the current WGS and FGS predictions. To identify genes absent from the current WGS and FGS predictions, all reads were used to build empirical transcript models. The resulting dataset contains 31,015 models longer than 100 bp that are completely intergenic relative to the existing WGS gene models and are detected above 0.1 FPKM (Additional file 3). However, 27,685 of these intergenic models (89.3% of the total) were classified as TE-related or other repeat-related via BLAST, using previously validated parameters [41], or via RepeatMasker (see Materials and methods) (Table S3C in Additional file 3). A small number of these repeat-related transcripts (1,174; 4.2%) overlap with lncRNA loci [38]; a larger percentage of the non-repeat-related intergenic gene models (648 out of 3,330; 19.5%) show lncRNA locus overlap (Table S3A in Additional file 3).

Thus, most of the 3,330 non-repeat-related intergenic transcript models (Table S3B in Additional file 3) represent potential novel protein-coding genes. Although many of these models are small (100 to 200 bp, possibly incomplete transcripts), the overall average is 546 bp, with lengths extending up to 3.4 kb. The largest category of these did not show enriched expression in any one tissue (Table 2). However, both embryo sac and pollen samples were associated with significantly higher counts of tissue-enriched (that is, two-fold higher than any other sample) intergenic transcript models than either sporophytic sample assessed. Non-enriched, ES-enriched, and MP-enriched transcript models show a similar likelihood to encode proteins, based on BLAST and InterProScan assessment (ES, 25.9%; MP, 24.2%; non-enriched, 29.8%; Table S3A in Additional file 3).

**Table 2:** **Novel gene models identified by transcript assembly from RNA-seq data**

	**Non-TE/repeat-related gene models**	**Average length (bp)**	**TE/repeat-related gene models**	**Average length (bp)**
Seedling enriched (2× higher than other three tissues)	26	515	74	816
Pollen enriched (2× higher than other three tissues)	376	679	1,133	836
Embryo sac enriched (2× higher than other three tissues)	622	484	4,395	881
Ovule (without embryo sac) enriched (2× higher than other three tissues)	37	643	67	1,486
Not specific to any one tissue	2,269	540	22,016	939

Transcripts from TE-related sequences were detected at a higher level in gametophytes than in sporophytic tissues (Table 2), consistent with results in *Arabidopsis* [36]. This is despite filtering out a large number of repeat-matching reads prior to transcript assembly. To further assess this trend, gene models in both the FGS and the WGS annotated as 'probable transposon' were evaluated for expression in different tissues. Analysis of RNA-seq reads mapped to these gene models revealed that the gametophytes (particularly the embryo sac) are significantly more likely than the sporophytic tissues to express one of these (Table 3). A similar bias is found when focusing on gene models enriched in one tissue (defined as a two-fold signal increase over the other three tissues), with the set of ES-enriched genes overrepresented for 'probable transposon' genes compared with the two sporophyte samples and the total gene set.

**Table 3:** **Percentage of genes annotated as probable transposon genes expressed in gametophyte and sporophyte samples**

	**Probable transposon genes in the filtered gene set expressed above 0.1 FPKM (1.2% of total (456/39,635))**	**Probable transposon genes in the working gene set expressed above 0.1 FPKM (3.3**% **of total (3,692/109,324))**
All seedling expressed genes	0.9% (259/27,564)	1.3% (426/33,528)
All pollen expressed genes	1.0% (143/14,591)	1.4% (251/17,314)
All embryo sac expressed genes	1.1% (308/28,489)	2.3% (964/42,672)^S,P,O^
All ovule (without embryo sac) expressed genes	1.0% (263/26,338)	1.6% (560/35,727)
Seedling enriched (2× higher than other three tissues)	0.7% (58/8,066)	0.8% (65/8,335)
Pollen enriched (2× higher than other three tissues)	1.3% (30/2,224)^S^	2.3% (82/3,526)^S,O^
Embryo sac enriched (2× higher than other three tissues)	1.6% (83/5,011)^S,o,T^	4.4% (315/7,097)^S,P,O,T^
Ovule (without embryo sac) enriched (2× higher than other three tissues)	1.1% (19/1,751)	1.5% (85/5,475)
Dual Gametophyte enriched (pollen and embryo sac each 2× higher than both sporophyte tissues)	2.0% (12/591)^S,o,t^	2.3% (10/434)^S^

^S^Higher than equivalent seedling frequency at *P* ≤ 0.01.

^P^Higher than equivalent pollen frequency at *P* ≤ 0.01.

^O^Higher than equivalent ovule frequency at *P* ≤ 0.01.

^o^Higher than equivalent ovule frequency at *P* ≤ 0.05.

^T^Higher than total in gene set at *P* ≤ 0.01.

^t^Higher than total gene set at *P* ≤ 0.05.

### Validation of RNA-seq by quantitative RT-PCR

**Figure 3:**
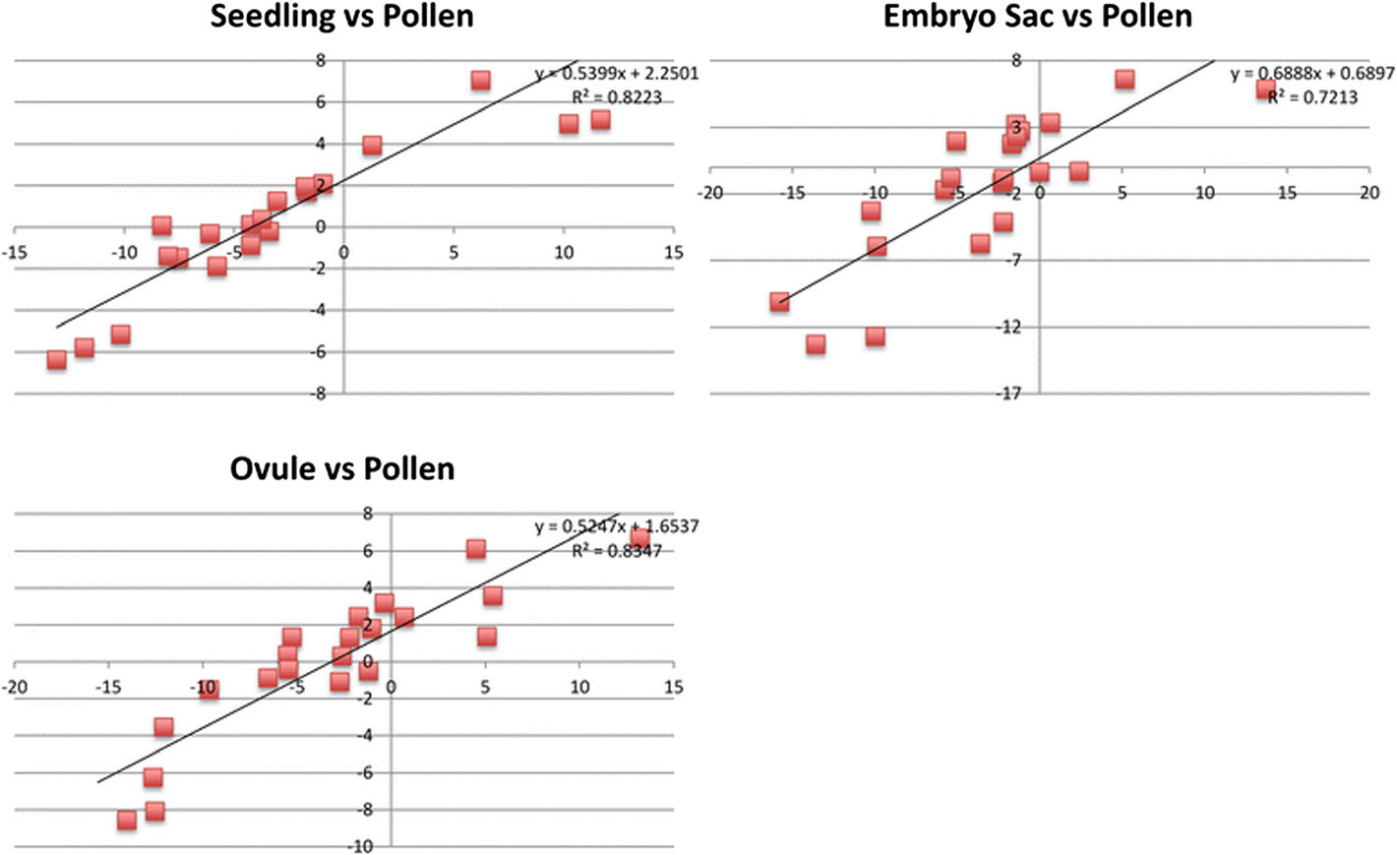
**Verification of RNA-seq with qRT-PCR.** The log_2_ (expression relative to actin and ubiquitin genes in test tissue by qRT-PCR/expression relative to actin and ubiquitin genes in pollen by qRT-PCR) is plotted on the y-axis and the log_2_ (expression in test tissue by RNA-seq(FPKM)/expression in pollen by RNA-seq(FPKM)). Although the slopes of the lines are not equal to one, the R^2^ values show good correlation between measurements using both types of data with the lowest correlation for the embryo sac samples. For RNA-seq values expression values were from B73 pollen, B73 seedling, combined W23-B73 embryo sac, and combined W23-B73 ovules without embryo sacs. For RT-PCR expression values, only W23 embryo sac and ovules without embryo sacs were used.

To verify the differential expression detected by the Illumina RNA-seq data, quantitative RT-PCR (qRT-PCR) was performed on a new set of three replicates for all four tissue types. A set of 46 genes was chosen randomly, based on the availability of *Ds* insertion alleles (see below). These genes had a range of average expression levels for each tissue: 0.16 to 537 FPKM for seedling; 0 to 6,635 FPKM for MP; 0.03 to 815 FPKM for Ov; and 0 to 417 FPKM for ES samples. One concern of the RNA-seq analysis was that amplification of cDNA of the ES and Ov samples prior to Illumina library construction may have introduced biases in composition of the library. To test the potential for biases, cDNA was prepared for all four tissues using similar quantities of RNA as was used in the original ES and Ov samples. The cDNA from these samples was then amplified prior to qRT-PCR. The qRT-PCR analysis from these samples was then compared with the RNA-seq expression data for all four tissue types. To corroborate the expression levels measured by RNA-seq, the ratio of expression levels between tissues using RNA-seq was compared with the ratio of expression as measured by qRT-PCR. For all genes, expression of genes in seedling, Ov, and ES were measured relative to their expression in MP, since MP is the least complex tissue of the four samples on a cellular level. As can be seen from the R^2^ values (0.83, 0.82 and 0.72), the ratios of gene expression measured between tissues by RNA-seq and by qRT-PCR are highly correlated (Figure 3). The lowest correlation was seen with the ES samples, which had the least amount of starting material for RNA-seq, suggesting that there is some loss of fidelity with amplification from small amounts of starting RNA. However, these validation experiments support the reliability of the relative values provided by the RNA-seq analysis.

### Comparison of gametophytic and sporophytic gene expression programs

Comparison of the lists of FGS gene models above an average expression threshold of 0.1 FPKM in each sample type (Additional file 4) revealed a number of features (Figure 1G). The largest set, 12,062 genes, shows expression above the threshold in all four tissue types. Seedling had the highest percentage of genes in its transcriptome above 0.1 FPKM that are not shared with any of the other samples at 8.4%, compared with ES samples at 6.0%, MP at 5.0%, and Ov with the lowest frequency of unique genes at 1.9%. The lower numbers of unique genes for Ov are not surprising given that the ES samples also contain small amounts of contaminant nucellus cells. Corroborating earlier studies on maize pollen mRNA diversity [42], and similar to *Arabidopsis* [33], the MP transcriptome is the least complex of the four with half as many of the FGS genes expressed above a threshold of 0.1 FPKM as the other tissues (Table 2). This is consistent with the view that MP is highly specialized compared with the other three tissue types, as 10,662 genes shared by the other three tissue types are not detected in MP (Figure 1G). Thus, a picture emerges of a relatively large core of genes expressed across all four developmental stages, with functional specialization potentially due to the combination of differences in expression level for this core set, plus developmentally specific expression of a smaller set of genes.

**Figure 4:**
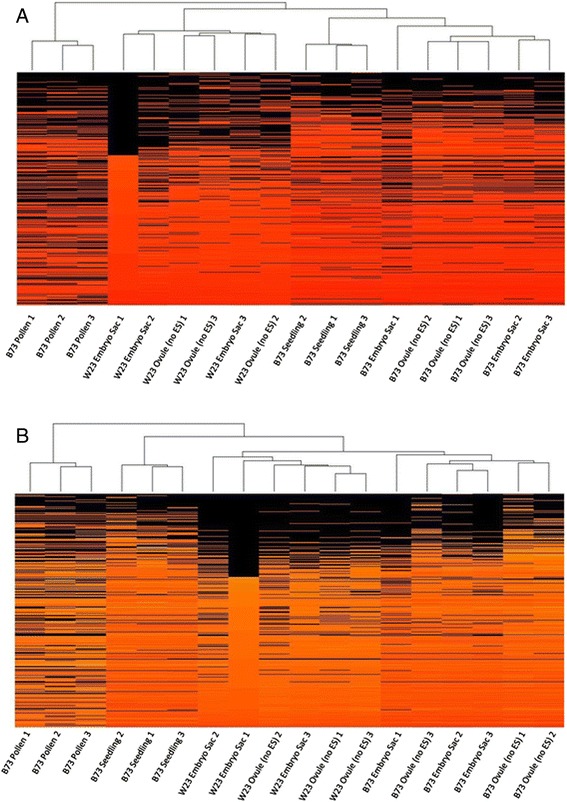
**Hierarchical clustering of replicates based on expression profiles using the R statistical package.** Genes are organized vertically based on expression in W23 ES sample 1. **(A)** Clustering based on FGS gene expression FPKM except for genes with 0 FPKM in all 18 samples. **(B)** Same as in (A) except that genes having 0 FPKM in all six W23 ES and Ov samples but having reads above 0 in the B73 ES or Ov samples were also omitted to remove possible artifacts caused by read mapping difficulties.

To determine the similarity of the transcriptomes of different tissues to one another, including between different inbred lines (B73 and W23), hierarchical clustering was used to compare the 18 replicates across 6 tissues and/or genotypes (Figure 4). The first analysis used all FGS genes, only excluding genes that were below threshold in all samples (Figure 4A). Due to the possibility that polymorphisms between W23 and B73 could lead to inaccurate measurement of expression of some W23 genes, a second comparison was also made. This second analysis (Figure 4B) excluded the approximately 6,000 genes for which no reads were detected in any of the six W23 (ES and Ov) samples. As in *Arabidopsis* [14], these analyses supported the view that the MP transcriptome is the most distinct, clustering away from the other samples regardless of which gene set was used.

### Relationship between gene expression pattern and duplicate gene retention

The maize genome consists of two subgenomes as a consequence of an ancient allotetraploidy event, and thus genes in the modern genome can be classified as either singletons (if the corresponding homeolog has been lost since tetraploidization), or duplicates (if both genes have been retained) [11]. Subgenome 2 is characterized by higher gene loss and lower gene expression of retained genes than subgenome 1. To determine if expression in the gametophytes is distributed differently in the two subgenomes, two sets of gene lists were developed for each sample type from B73. The first set (the total transcriptomes) included all FGS gene models above a threshold of 0.1 FPKM in that sample type; the second set (the tissue-enriched transcriptomes) included only those gene models from the total transcriptomes that were at least two-fold higher in a sample type relative to all three other sample types (see Materials and methods). These gene model lists were then mapped to high-confidence subgenome 1 and 2 sets [11] (Table 4). As expected, in all four tissues the percentage of genes expressed above the threshold is higher for subgenome 1 than subgenome 2. However, for both the total MP transcriptome and the MP-enriched gene list, the percentage of genes in subgenome 2 is significantly higher than it is for the total gene list, or for the other tissue-focused gene lists. None of the other tissue transcriptomes show overrepresentation of subgenome 2 compared with the whole genome. A breakdown of how the tissue-enriched genes are distributed in the subgenomes (as either singletons or part of a retained duplicate pair) reveals the basis for this difference (Table 5; Additional file 5). Relative to the other expression categories, and to the entire FGS set, MP-enriched genes are more likely to be duplicates, retained in both subgenomes. Furthermore, the MP-enriched set has a significantly lower distribution of subgenome 1 singletons, supporting the idea that this set of genes is less likely to have lost subgenome 2 homeologs. Finally, when focusing on the retained duplicate pairs in the four tissue-enriched gene sets, the MP set has a significantly greater proportion of pairs in which both members are represented; in fact, one-quarter of the gene models in the MP-enriched set that could be assessed via subgenome mapping are a member of an expressed pair. These data are consistent with the idea that pollen places some exceptional requirement on gene function, such that selection pressure results in retention and expression in pollen of a higher proportion of both genes of a duplicate pair.

**Table 4:** **Expression of subgenome 1 and subgenome 2 assigned genes in gametophyte and sporophyte samples**

	**Subgenome 2 to subgenome 1 ratio (63.1% for all subgenome2/all subgenome1 (7,118/11,282))**
All seedling expressed genes	62.6% (6,047/9,657)
All pollen expressed genes	67.5% (3,421/5,066)^s,e,o,t^
All embryo sac expressed genes	63.3% (5,820/9,195)
All ovule (without embryo sac) expressed genes	63.6% (5,541/8,716)
Seedling enriched (2× higher than other three tissues)	57.3% (1,749/3,054)
Pollen enriched (2× higher than other three tissues)	73.0% (465/637)^s,t^
Embryo sac enriched (2× higher than other three tissues)	63.4% (645/1,017)
Ovule (without embryo sac) enriched (2× higher than other three tissues)	62.7% (207/330)

^s^Higher than equivalent seedling frequency at *P* ≤ 0.01.

^e^Higher than equivalent embryo sac frequency at *P* ≤ 0.05.

^o^Higher than equivalent ovule frequency at *P* ≤ 0.05.

^t^Higher than total gene set at *P* ≤ 0.05.

**Table 5:** **Tissue-enriched gene models mapped to singletons and duplicates in the maize subgenomes**

	**Singleton - subgenome 1**	**Singleton - subgenome 2**	**Duplicate - subgenome 1**	**Duplicate - subgenome 2**	**Total number mapped to subgenomes**	**Number of duplicate pairs, both represented in the enriched set (percentage of set)**
Seedling enriched (2× higher than other three tissues)	41.0% (1,369)	16.9% (564)	21.3% (712)	20.8% (693)	3,338	338 (20.3%)
Pollen enriched (2× higher than other three tissues)	32.6% (259)	17.9% (142)	23.1% (184)	26.4% (210)	795	99 (24.9%)
Embryo sac enriched (2× higher than other three tissues)	39.4% (443)	17.6% (198)	21.9% (246)	21.2% (238)	1,125	55 (9.8%)
Ovule (without embryo sac) enriched (2× higher than other three tissues)	43.4% (155)	17.4% (62)	20.7% (74)	18.5% (66)	357	8 (4.5%)
Filtered gene set	37.5% (4,860)	16.7% (2,161)	23.0% (2,982)	22.7% (2,945)	12,948	

For the 4 × 4 categorical comparison of the expression sets versus the subgenome mapping characters, the chi-square value is 25.88, for *P* < 0.005, indicating a significant difference in the distributions, due to the pollen set. No significant difference is present comparing only seedling, embryo sac and ovule sets (χ^2^ 2.61; *P* > 0.5).

### Gene Ontology functional category enrichment

Functional enrichment of GO terms was performed for lists of genes with particular expression patterns using the online Agrigo GO Analysis Toolkit [43] using the modified average expression values of the FGS genes. GO term overrepresentation was performed for the full transcriptome above 0.1 FPKM for each tissue, and for the tissue-enriched gene lists (Additional file 6; see Materials and methods for description).

Comparison of the GO terms overrepresented in the full transcriptome of each of the four tissue samples revealed that the MP samples had the most GO terms (114) that were not shared with the other samples (Additional files 7 and 8). The largest number of overrepresented GO terms were in the ES and Ov samples, but many of these were shared, with GO terms unique to the ES in large part related to the DEFENSIN/LURE (DEFL) family (see below). By far the largest group of GO categories overrepresented in each of the four full transcriptome gene lists was shared by all four samples (212 GO terms), followed by the number shared by ES, Ov, and seedling (90 GO terms). Thus, the overall analysis suggests that the distinctiveness of the pollen transcriptome is extended to the functional level.

Second, analysis of overrepresented GO terms was performed for tissue-enriched gene lists to identify potential tissue-specific functions (Additional file 9). A dual gametophyte-enriched gene set (enriched in both MP and ES, relative to both Ov and seedling) was also identified. The Ov sample had the fewest overrepresented GO terms of all four tissue-enriched gene lists, with apoptosis-related terms being significantly increased (Table S8D in Additional file 9). In seedling genes, GO categories related to photosynthetic functions and environmental responses were overrepresented (Table S7A in Additional file 6 and Table S8A in Additional file 9).

For the MP-enriched genes, the most significantly overrepresented GO categories include functions related to the actin cytoskeleton, and GO terms potentially related to pollen tube growth and penetration of the pistil (for example, the cell wall-loosening expansins; pectinesterases and glycosidases; Table S8B in Additional file 9). Additionally, there is significant overrepresentation of post-translational protein modification, driven in large part by an abundance of protein kinases. A few members of the DEFL family are also specifically overrepresented in the MP transcriptome. MP-enriched genes in subgenome 2 (Tables 4 and 5), were also examined for overrepresented GO terms (Additional file 10). In this subset of subgenome 2 genes, functions related to localization and transmembrane transport, as well as pectinesterase activity, were the most significantly overrepresented GO terms.

For the ES, biological processes and molecular functions related to transcriptional regulation were the most highly overrepresented (Table S8C in Additional file 9). Interestingly, as in the MP transcriptome, expansin gene expression is significantly overrepresented in the ES transcriptome, although a different set of expansins from those found in MP. These genes may facilitate the rapid expansion of the embryo sac within the surrounding nucellus. The other most significant GO terms in the ES-enriched genes include nucleotide metabolic processes. Enrichment of this category is entirely driven by the high number of ES-enriched members of the DEFENSIN/LURE (DEFL) family, as these small proteins contain a knottin fold with a dinucleoside diphosphate kinase core. The shared gametophyte-enriched gene set shows similar GO category overrepresentation, again driven by DEFL proteins. Thus, in total, three different sets of DEFL genes were found, each overrepresented among the transcripts showing that expression character: ES-enriched; MP-enriched; and dual gametophyte-enriched. Some members of this family have previously been shown to be expressed in synergids in maize and to function as pollen tube attractants in *Torenia* [28,44-47].

### Analysis of transcription factor gene families

Because transcriptional regulation GO terms were significantly overrepresented in the ES-enriched gene list, all known TFs in maize from the Grass Transcription Factor Database [48,49] were assayed for tissue-enriched expression in the gametophyte and sporophyte tissues (Additional file 11). Using this more comprehensive TF list, significant overrepresentation for the aggregate of all TF families was detected in the embryo sac. Five separate TF gene families showed significant overrepresentation (*P* < 0.05) in the ES-enriched gene list (in order of significance, AP2-EREB, WRKY, MYB-RELATED, NAC, and MADS-box), and no TF families were significantly under-represented in the embryo sac. This contrasts with MP, seedling, and Ov, where there was a global underrepresentation of TFs. In the MP-enriched gene list, only orphan TF genes and MADS box genes (including a previously identified MADS box gene specific to pollen [50]) are overrepresented. Neither seedling nor Ov had any TF gene families overrepresented by the criteria used.

Because the MADS gene family appeared in both MP and ES gene lists, it was analyzed in greater detail (Additional file 12). Ten MADS genes are in the MP-enriched gene set, and four are present in the dual gametophyte-enriched gene set (although three of these four are significantly higher in MP than ES and so are also present in the MP-enriched set). Enrichment for different MADS family members in the ES and the MP is reminiscent of the distribution of ES-specific and MP-specific MADS genes in *Arabidopsis* [17,27,51-54]. In *Arabidopsis*, MIKC* MADS genes are overrepresented in the MP [13,15,55], whereas the type I class α and β genes are overrepresented in the ES [53]. In maize, all MIKC* MADS genes are enriched in MP, supporting the conclusion for an ancient role for these genes in the male gametophyte [56]. Other MP-enriched maize MADS genes fall into the MIKC and the type 1 class α groups. Maize ES-enriched genes fall into the MIKC and the type 1 class α and γ groups, which is somewhat distinct from the pattern in *Arabidopsis*. There are no clear type 1 class β MADS genes in maize, just as there are none reported in rice [57].

The phylogenetic relationships between ES-enriched genes were also determined for the other TF families overrepresented in the embryo sac. The NAC gene family was particularly striking, with 25 of 25 genes in one clade being ES-enriched and only one of the 109 genes in the other clade being ES-enriched (Additional file 13). For the AP2-EREB, WRKY and MYBR families, ES-enriched genes were broadly distributed across most clades, although differences between subgroups exist (for example, local over-representation of a few closely related genes; Additional files 14, 15, and 16). For many of these subfamily enrichments of TFs, the shared ES expression patterns are associated with syntenic regions, rather than tandem duplications, and may reflect an ancestral embryo sac function for these branches of the gene family.

### Analysis of small peptide gene family expression

**Figure 5:**
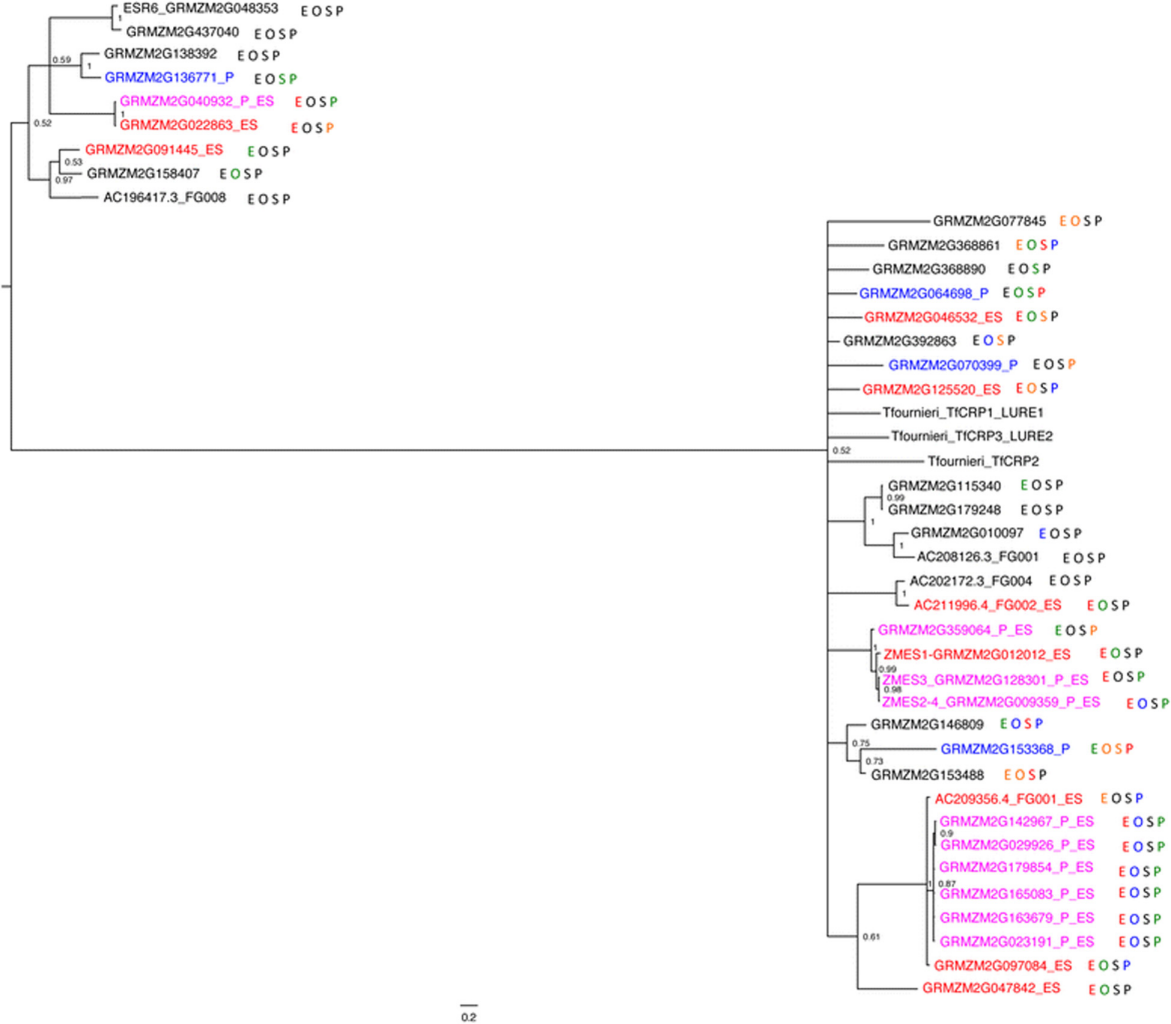
**Phylogeny and expression of maize DEFL genes.** Gene names in blue are part of the MP-enriched gene set. Gene names in red are part of the ES-enriched gene set. Gene names in magenta are part of the dual gametophyte-enriched gene set. Expression levels are indicated by color of the letter of each sample type with red meaning >10 FPKM, orange between 1 and 10 FPKM, green between 0.1 and 1 FPKM, blue greater than zero but less than 0.1 FPKM, and black having 0 reads. E, embryo sac expression; O, ovule without embryo sac expression; S, seedling expression; P, mature pollen expression. *Torenia* LURES are included for reference. Posterior probability values are given at node positions.

**Figure 6:**
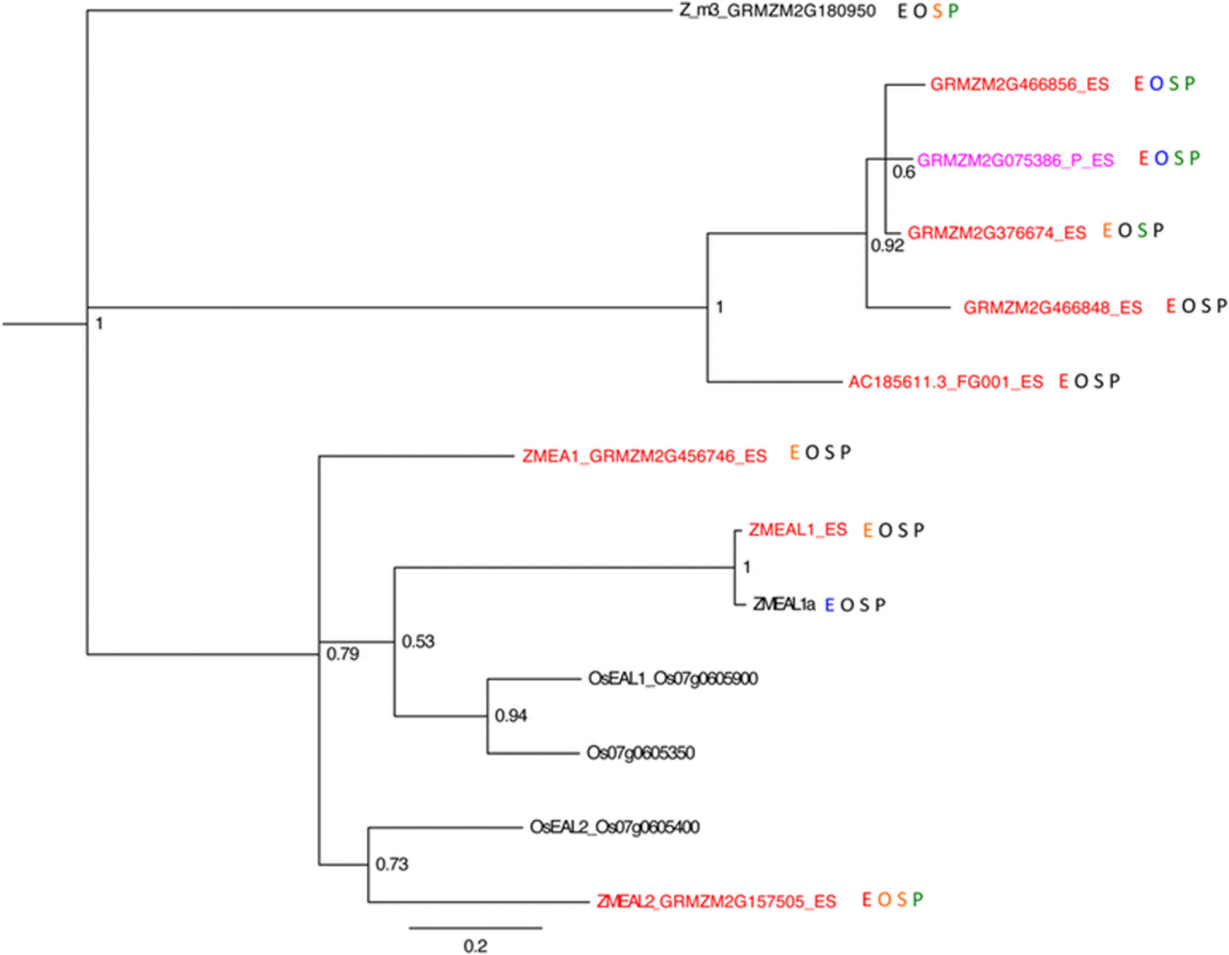
**Phylogeny and expression of maize EAL genes.** Gene names in blue are part of the MP-enriched gene set. Gene names in red are part of the ES-enriched gene set. Gene names in magenta are part of the dual gametophyte-enriched gene set. Expression levels are indicated by color of the letter of each sample type with red meaning >10 FPKM, orange between 1 and 10 FPKM, green between 0.1 and 1 FPKM, blue greater than zero but less than 0.1 FPKM, and black having 0 reads. E, embryo sac expression; O, ovule without embryo sac expression; S, seedling expression; P, mature pollen expression. Rice genes from Krohn *et al*. [64] are included for comparison. Posterior probability values are given at node positions.

The expression pattern of small peptide gene families was investigated in greater detail based on three reasons: (1) GO analysis highlighted small peptide DEFL genes as overrepresented in all three gametophyte-enriched gene sets; (2) shorter transcripts were more prevalent in the ES transcriptome compared with the other three tissues (data not shown); and (3) probes for small peptide genes were often omitted from earlier microarray studies. Characterization focused on two families with known gametophyte members: DEFENSIN/LURE (DEFL) [44], and Zm Egg Apparatus1 (ZMEA1)-LIKE (EAL) [58-61]; and two families that had not previously been shown to have gametophyte-expressed members, CLAVATA3-ESR (CLE) [62], and LITTLE ZIPPER (ZPR) [63] (Figures 5 and 6; Additional files 17 and 18).

Four maize DEFL genes (*ZmES1*, *2*, *3*, and *4*) had previously been identified in the A188 inbred line, and characterized as embryo sac-expressed [28]. In the B73 genome these four genes correspond to three tandemly duplicated genes, which we have termed *ZmES1*, *ZmES3*, and *ZmES2/4*. One likely explanation for the discrepancy is that an additional duplication exists in A188. Using BLAST to identify similar genes in the B73 genome identified 39 DEFL gene models in the B73 v5a WGS, a larger DEFL family than in the AgriGO database. Expression analysis shows clear bias for expression of these genes in the embryo sac. Twenty of these are expressed above 1 FPKM in ES, compared to five above 1 FPKM in MP, six in seedling, and four in Ov. In all, 23 of the 39 DEFL genes have tissue-enriched expression in one or both of the gametophytes. The strong embryo sac enrichment for DEFL gene expression contains genes in three clades within the DEFL family, one group including *ZmES1* through *ZmES2/4*, one clade with all members in either the ES-enriched or dual gametophyte-enriched gene set, and a third clade more divergent from the rest of the DEFL genes, including the endosperm-expressed *ESR6* gene (Figure 5). Many of the ES-enriched DEFL family members are found in tandem clusters of recently duplicated family members, as exemplified by the *ZmES1-3-2/4* cluster. The relationships within these clusters are more robust than those between the less recently diverged groups. Of the 19 DEFL genes in the ES, 10 are also dual gametophyte-enriched, although the level of expression in the MP is consistently lower than in the embryo sac.

The EAL family was founded by the maize embryo sac-specific gene *Zm Egg Apparatus1* (corresponding to GRMZM2G456746), which encodes a protein that functions in the embryo sac as a pollen tube attractant [58]. This family is characterized by an EA1 box near the carboxyl terminus [59]. Three additional small peptide EAL genes have been described: *ZMEAL1* (transcript maps upstream of and includes GRMZM2G576769) [64], *ZMEAL2* (GRMZM2G157505) and GRMZM2G180950. BLAST querying for other small peptide *ZmEA1* homologs in the B73 genome identified six additional genes (Figure 6). *ZMEAL1* is expressed in the embryo sac and required for normal antipodal cell development [64]. Like the DEFL family, *EAL* genes also show family-wide enrichment in the embryo sac, with eight above 1 FPKM in ES, none in MP, two in seedling, one in Ov, and one with expression below 1 FPKM in all tissues tested. *ZMEA1*, *EAL1*, and *EAL2* are part of a cluster of four tandemly duplicated genes on chromosome 7 with the fourth gene adjacent to and nearly identical to *EAL1* but with much lower expression. All members of this cluster are preferentially expressed in the embryo sac, albeit at different levels. A second clade including four tandemly duplicated *EAL* genes located on chromosome 8 also has every member in the ES-enriched gene set.

In contrast, the CLE and ZPR families do not show family-wide enrichment for embryo sac expression. Twenty-six and eight genes were identified for the CLE and ZPR families, respectively (Additional files 17 and 18). For the CLE family there were nine above 1 FPKM in ES, two in MP, seven in the seedling, and four in Ov. The CLE family was almost completely absent in MP, with 24 of the 26 members having no reads in the MP. For the ZPR family there were two above 1 FPKM in ES, one in MP, three in seedling, and one in Ov. Some of the ZPR family members are characterized by low expression in the ovule and no expression in the ES, suggesting they are expressed in portions of the ovule excluded from the ES samples (for example, integuments). The expression of small peptides in the gametophytes is therefore not a general phenomenon; rather, the DEFL and EAL families are likely enriched in these tissues for critical roles in gametophyte biology.

### Test of gametophyte-expressed genes for gametophyte function

Genes expressed in the gametophytes should be enriched for genes with gametophyte-critical functions. Such a function can be confirmed by observing reduced transmission of a mutation in that gene through the relevant gametophyte to the next generation. This reduced transmission is also predicted to result in reduced recovery of mutations in gametophyte essential genes. Thus, there should be a bias against recovering mutations in the sets of MP-enriched and ES-enriched genes compared with sporophyte-enriched genes identified in this study. The large collections of sequence-indexed transposon insertions for both the *Mutator* (*UniformMu* and the Photosynthetic Mutant Library [65-67]) and *Ac*/*Ds* [68] systems available in maize allowed a test of this prediction.

A baseline for transposon insertion rates in these collections was generated by assessing the frequencies for insertions into particular regions of the gene models of the FGS and the RGS. The regions were assessed separately given the known bias in certain transposon insertion patterns (for example, *Mu* is targeted near transcription start sites [69]), and the presumed likelihood of affecting gene function (for example, exons in coding sequence versus introns). As expected, the FGS was associated with significantly higher rates of TE insertion than the RGS (which is also associated with significantly higher methylation [70]) in the insertion collections assessed (Additional file 19). The higher methylation may be associated with a relative decrease in accessibility for these sequences, and thus a decrease in transposon insertion rates [69]. Notably, a set of FGS gene sequences identified as containing TE/repeat sequences (see Materials and methods) was also associated with a bias toward fewer insertions relative to the non-TE FGS gene models (Additional file 19). Therefore, these TE/repeat-related gene models in the FGS were left out of further analyses of insertion frequency.

The frequency of associated *Mu* and *Ac/Ds* insertions was then calculated for the seedling, mature pollen and embryo sac sets of tissue-enriched gene models (Table 6; Additional file 20). The *UniformMu* population is the largest currently available, with 41,543 flanking sequence locations (April 2012, release 5); in addition, the propagation scheme for this population relies on self- and sib-pollination, imposing selection against both male and female gametophytic functions. Consistent with the predicted bias, in this population *Mu* insertions into the MP-enriched and ES-enriched gene sets were significantly less common relative to the seedling gene set. The decreased prevalence of *Mu* insertions could not be explained solely by differences in gene size among the gene sets, as the bias remains detectable when normalized based on average size in base pairs for each region (Table 6). Although flanking sequence data for the Photosynthetic Mutant Library population (May 2013) is only approximately one-fourth that available for *UniformMu*, a similar, significant decrease in *Mu* insertions for the MP-enriched and ES-enriched sets is also discernible in this population (Additional file 20). Notably, the deficit appears to be strongest in the MP-enriched gene set for insertions in exons in both populations, consistent with an effect associated with gene function. For the ES-enriched gene set, the strongest decreases appear to be in introns and the proximal promoter, in addition to exons, suggesting that factors in addition to gene function play a role in influencing insertion likelihood.

**Table 6:** **Reduced frequency of insertion mutants in gametophyte- versus seedling-enriched genes**

**Expression characteristic**	**Total gene models tested**	**Percentage of gene models tested with a coding sequence** ***Ac/Ds*** **insertion**	**Percentage of gene models tested with a coding sequence** ***Mu*** **insertion**
Seedling-enriched genes	7,385	1.6% (0.70%)	19.9% (9.3%)
Pollen-enriched genes	2,042	1.9% (0.90%)	12.7% (6.1%)*
Embryo sac-enriched genes	4,238	0.8% (0.43%)*	13.2% (7.1%)*

Percentages in parentheses show the frequency of insertion per gene normalized for gene size. *Significantly lower than frequency of insertions in seedling enriched genes, *P* < 0.01.

The smaller number of available mapped *Ac/Ds* insertion locations limits the power to detect bias, but the largest population available (*Ds* Mutagenesis, 1,969 flanking sequence locations) is a useful comparison to the *Mu* populations as new insertions are selected and propagated solely through the female. Therefore, male-specific gametophytic insertions should not be selected against in this population, in contrast to insertions in female gametophyte genes. Consistent with this prediction, a significant bias against *Ac/Ds* insertions is found associated with the ES-enriched gene set in exons, introns and the 3′ end of predicted transcripts. Further, no significant difference in insertion bias was found between the seedling- and MP-enriched gene sets. However, the MP-enriched gene set is approximately half the size of the ES-enriched gene set, raising the possibility that the limited size prevents a robust assessment of any differences.

To address gametophyte function among these gene sets more directly, we also examined a set of 27 *Ds* insertions from the *Ds* Mutagenesis population in genes with a range of expression levels to see if the expression pattern would predict whether or not they would have transmission defects (Table 7; Additional file 21). Heterozygous plants carrying the mutations were crossed reciprocally with homozygous wild type and their progeny tested for the presence of the *Ds* insertion using PCR. Transmission of the *Ds* insertion was called as reduced if the frequency in progeny was significantly less than 50% using a χ^2^ test with a cutoff of *P* < 0.05. Nine of the genes had highest expression in the MP (eight of these were in the MP-enriched set), eight of the test genes had highest expression in the embryo sac (four of them in the ES-enriched set), and the remaining ten genes had their highest expression in one of the sporophyte samples (six in one of the sporophyte-enriched sets). Note that the analysis of transposon insertion patterns shows that this population (Tables 7; Additional file 21) is biased against recovery of *Ds* insertions in genes highly expressed in the embryo sac; due to the propagation scheme for this population, mutations with strong female transmission defects are likely to be systematically excluded. All 27 mutations were tested for transmission as females; two of the eight *Ds* insertions in the genes with highest expression in the ES had slightly reduced transmission through the female, whereas none of the other 19 tested had reduced female transmission. Twenty-two were tested as males; of these, 9 were in the MP-enriched list and 13 were not. Two of the nine mutations in MP-enriched genes had significantly reduced pollen transmission, whereas none of the other 13 did. Notably, the two mutations with reduced male transmission were in genes likely associated with cytoskeletal and signaling functions crucial for pollen: *profilin3* [71] and a potential calcium-binding (C2 domain) protein. The roles of the two genes associated with the female transmission defects, encoding a RING finger protein and a hypothetical protein, are less clear. Taken together, four of 17 tests of mutations in genes with highest expression in one of the gametophytes showed reduced transmission through that gametophyte, whereas none of the 32 tests without gametophyte enrichment of expression showed reduced transmission through that gametophyte, confirming that the probability of a gene being required for function in the gametophyte can be predicted on the basis of the relative expression between tissues.

**Table 7:** **Transmission frequency of** ***Ds*** **insertions in genes with high and low gametophyte expression**

	***Ds*** **insertions with reduced transmission**
Female transmission of *Ds* insertions in genes with highest expression in embryo sac	2/8
Male transmission of *Ds* insertions in genes with highest expression in pollen	2/9
Transmission through the opposite gametophyte for genes with highest expression in the embryo sac or pollen	0/16
Transmission through either gametophyte for genes with highest expression in one of the sporophyte tissues	0/16 (or 1/16*)

Reciprocal crosses were made between wild-type W22 plants and plants carrying *Ds* insertions in genes with varying expression patterns. *Ds* insertions that were recovered in fewer than 50% of the progeny (*P* < 0.05) were scored as having reduced transmission. See Additional file 21 for supporting data.

*One of these *Ds* lines is on the borderline of being significantly lower than 50% (*P* = 0.0474).

The frequency of *Ds* insertions with reduced transmission is significantly higher for genes with the highest expression in the gametophyte tested (4/17) than other genes (Fisher’s exact test *P* = 0.011 for 0/32 non-gametophyte genes, and Fisher’s exact test *P* = 0.043 for 1/32 non-gametophyte genes).

## Discussion

The ultimate function of the gametophyte is the production of viable offspring through the fusion of the male and female gametes. The process of double fertilization is unique to flowering plants and results in the formation of a diploid (one maternal: one paternal) embryo and typically triploid (two maternal: one paternal) endosperm. Similarities between the male and female gametophytes may result from conserved functions in gamete production or may have arisen from the inheritance of an ancestral condition of bisexual gametophytes found in many non-seed plants (for example, *Physcomitrella*) [72]. However, the developmental patterns and cellular functions of the gametophytes are quite distinct. Identification of the genes active in the gametophyte generation provides a better understanding of their function, similarities, and uniqueness. To better understand the function of the maize gametophyte generation we have performed a full transcriptome analysis of mature male and female gametophytes using RNA-seq.

Genome-wide expression analysis reveals several implications for maize genome organization. Analysis of expression of genes annotated as transposon-related, as well as analysis of intergenic transcript models with similarity to repeat sequences, reveals that repetitive DNA elements are more likely to be expressed in both the male and female gametophytes than in sporophytic tissues. These data agree with results in *Arabidopsis* that gametophytes produce RNA from highly repetitive DNA elements [23,27,36]. Perhaps, as in *Arabidopsis*, in maize this is done as a means for silencing mobile elements in the germline, although the data here do not resolve in which cells these transcripts accumulate or are synthesized. Future experiments are necessary to determine if these transcripts are present in the gametes, whether or not they are transcribed in the gametes themselves, or if, as is the case in *Arabidopsis* pollen, they are transcribed in subsidiary cells (that is, the antipodal cells and synergids of the female gametophyte and the vegetative cell of the pollen grain). Expression of repetitive elements is not identical between the male and female gametophytes, with a greater likelihood for their expression in the female than in the male.

In *Arabidopsis* central cells, non-exonic transcripts, including known transposon and other intergenic transcripts, are more common than in other tissues - approximately two- to four-fold more non-exonic transcripts are in central cells than in seedlings or immature floral buds [34,73,74] - raising the possibility that transcriptional activity in ‘intergenic’ regions is a common feature of angiosperm gametophytes. *Arabidopsis* pollen also has a high frequency of intron reads [33] as well as expression of TEs [36]. In *Arabidopsis*, like maize, most predicted intergenic transcripts in the gametophytes are less than 500 bp [33]. However, most of the non-exonic *Arabidopsis* central cell reads were intronic, suggesting that this is driven in large part by incomplete annotation [34]. In contrast, in maize 90% or more of these reads are intergenic, suggesting that both incomplete annotation and TE transcripts are responsible for the exceptional ES transcriptome. Consequently, true intergenic transcriptional activity may vary between species. The higher expression of transposons and other intergenic sequences in maize embryo sacs may reflect either a higher activity of maize transposons than of those in *Arabidopsis* or the difference between sampling the whole embryo sac in maize versus the central cell in *Arabidopsis*. Cell-specific analysis of these transcripts in maize is needed to resolve whether it is one of these two alternatives or a combination of the two. Two classes of transposons are also expressed in rice ovules but it is not known if these are in the embryo sac or the surrounding ovule tissue [75].

Like the pattern of TE transcripts, intergenic, non-repeat transcripts are more common in ES samples than other tissues. Potential novel genes were defined as gene models assembled directly from the RNA-seq data that lacked homology to known TEs and other repeats. More potential protein-coding novel genes were identified in ES-enriched and MP-enriched gene sets than in sporophyte-enriched sets, with the greatest number present in the embryo sac. The relative inaccessibility of this tissue may have caused embryo sac-specific transcripts to be underrepresented in the expression data used to help build maize gene models, and thus be omitted from annotated gene sets. The high number of gametophyte transcripts intergenic to the WGS may be an additional consequence of the genome-wide relaxation of silencing of repetitive elements (and sequences adjacent to repetitive elements) in the gametophytes compared with the sporophyte. RNA-seq transcript assembly, including the samples in this study, identified lncRNA genes in the maize genome, and many of these were also found to be intergenic to WGS gene models [38]. Interestingly, reproductive tissues, including pollen and embryo sac, had more examples of lncRNA expression than any other tissues characterized.

The pollen transcriptome is also notable for its unusual representation in the two subgenomes of maize. Maize consists of two subgenomes from an ancient allotetraploidy event, with subgenome 2 characterized by reduced expression and reduced gene retention rates relative to subgenome 1 [11]. However, relative to the other three tissues assessed (which conform to expectations), pollen is associated with a significantly greater proportion of expression associated with genes of subgenome 2. This increase in subgenome 2 expression is not due to over-representation of pollen singleton genes in subgenome 2 (that is, genes for which the corresponding subgenome 1 duplicate has been lost over evolutionary time), but rather due to a retention of more duplicate pairs (that is, both subgenome 1 and 2 genes are retained in the genome) and correspondingly fewer pollen singleton genes in subgenome 1. Moreover, both members of a duplicate pair are more likely to be in the MP-enriched transcriptome than duplicates are to be in the other three tissues, consistent with the idea that expression of both plays a functional role in pollen. Thus, selection could be acting to maintain functional copies of both members of pollen-expressed genes following tetraploidization.

The gene balance hypothesis, which emphasizes that the expression dosage of genes encoding members of multi-subunit complexes, components of signal transduction pathways, or TFs needs to be maintained for correct function, has been invoked as an explanation for the retention of duplicates in genomes [76,77]. In one view, this balance would be even more critical in the male gametophyte, and therefore may result in a greater proportion of duplicate retention. First, the male gametophyte is haploid, so loss of one gene copy via mutation after tetraploidization reduces expression by half in the first generation, rather than by one-quarter, as would occur in the diploid. Second, differentiating it from the female gametophyte (which did not show such preferential retention), in an outcrossing species such as maize, pollen and the pollen tube are potentially under more stringent selection than other phases of the life cycle, via intense competition as a haploid for efficient pollen tube germination, tip growth and fertilization processes. Consistent with this idea, pollen-specific genes in an outcrossing relative of *Arabidopsis* (*Capsella grandiflora*) are associated with stronger purifying selection and greater proportion of adaptive substitutions than sporophyte-specific genes [78]. In this interpretation of gene balance, one would expect to see a larger percentage of pollen-critical genes to be retained as duplicates in maize, and furthermore, that mutation of either copy should result in a deleterious phenotype. At least one such example has already been described, the *rop2*/*rop9* duplicate pair, although the deleterious effect of *rop2* mutation is only revealed when competing with wild-type pollen [79]. This interpretation thus predicts that the MP-enriched duplicate genes identified herein are more likely to be associated with such competitive defects. Consequently, it also suggests that the overrepresented GO category processes identified in the MP-enriched subgenome 2 set (localization, transmembrane transport, and pectinesterase activity) are more likely subject to such dosage sensitivity.

Analysis of GO categories confirms previous results (for example, [10-13]) that regulation of a dynamic cytoskeleton is an important aspect of pollen biology. Additionally, post-translational modification is also overrepresented in the pollen transcriptome. Protein modification (for example, protein phosphorylation) may facilitate the rapid growth reorientations in response to local cues necessary for pollen tube function. In the ES-enriched gene set, regulation of transcription and small peptide DEFLs were overrepresented. Because of the presence of the DEFL gene family in the embryo sac transcriptome, additional small peptide gene families were also analyzed, since they were mostly not included in the GO term analysis. A second family of small signaling peptides, the EAL family, is also overrepresented in the ES transcriptome. Some members of both of these families have previously been shown to have female gametophyte expression [28,58], and to be involved in cell identity [64] and species-specific interactions with the pollen tube [47,60,61]. Here we have expanded the analysis of these gene families and shown that many members are enriched in the female gametophyte transcriptome. Certain DEFL genes show enriched expression in *Arabidopsis* central cells [34], suggesting that at least some of the DEFL enrichment reported here is associated with the central cell of maize. Correlations of gametophyte expression with phylogenetic relationships, including their location in tandem arrays, suggests that female gametophyte expression is an ancestral feature of some branches of both the DEFL and EAL gene families. The DEFL family also has members enriched in both male and female gametophyte transcriptomes. Mirrored expression of these small peptides in the two gametophytes may indicate a mechanism for reciprocal signaling between them. Shared and reciprocal signaling pathways of the male and female gametophyte will be easier to identify and resolve once it is known how cells perceive and respond to these small peptides.

Enrichment for transcriptional regulation in the embryo sac transcriptome was concentrated in five gene families: MADS, NAC, AP2/EREB, MYB-R, and WRKY. The MADS box gene family is also over-represented in *Arabidopsis* gametophyte transcriptomes. Maize and *Arabidopsis* both show a prevalence of pollen-expressed genes in the MIKC* family, suggesting that pollen function for MIKC* genes may predate the split between monocots and eudicots. Both maize and *Arabidopsis* also have members of the type 1 class α MADS genes. However, while in *Arabidopsis* the type 1 class β genes are overrepresented in female gametophytes, this clade is absent in maize. In maize, these functions may be taken over by other MADS gene clades (for example, the MIKC class, present in the ES-enriched gene set of maize, but not of *Arabidopsis*). The NAC, AP2, MYB-R, and WRKY gene families are also over-represented in the ES-enriched gene set. An overlapping set of TF families are over-represented in the transcriptome of whole rice ovules, including not only the AP2/EREB and MADS families but also the ABI3, AP2, YABBY, C2H2, HSF, LFY, MYB, and ZfHD families [75]. Many of these differences likely arise from the inability to compare the embryo sac to its surrounding ovule tissue in the rice study, but the shared groups may reflect gametophyte functions in the ancestor of maize and rice.

Mapping expression patterns on a gene phylogeny assists in evolutionary analyses, as a shared expression pattern by multiple members of a clade provides a hypothesis for the expression pattern of the common ancestor of that clade. The notable example of this is in the *NAC* TF family. A large ES-enriched clade includes duplicate genes from the ancestral maize allotetraploidization, as well as from older expansions of this gene family. In other cases, conserved genes with shared female gametophyte expression are part of a tandem cluster of genes with high similarity, suggesting more recent family expansion. This is seen for clusters of genes in the DEFL and EAL gene families, in which most or all of the genes in the cluster are expressed in the embryo sac. In fact, based on the phylogenetic analyses, the enrichment for female gametophyte expression of these families is apparently largely driven by expansion through tandem duplication. In some cases these tandem arrays are present in multiple grass lineages, as suggested by the maize *EA1* and *EAL1* genes being less similar to each other than to their rice homologs, which are also present as tandem duplications. In support of this hypothesis, the only one of the three rice *EA1*/*EAL1* genes in the cluster that was assayed by microarray hybridization was expressed in both the egg and synergids, supporting the model that gametophyte expression of these genes reflects shared ancestral gene regulation [80].

Analysis of mutants and mutant frequencies show that genes significantly enriched in the gametophyte transcriptomes are more likely to be required in the gametophyte than other genes. Mutant frequencies and transmission rates confirm that gametophyte-enriched expression is predictive of a requirement for gametophyte function without making additional accommodations for genetic redundancy. Consequently, the entire transcriptomic dataset is expected to prove useful for identification of candidates for gametophyte mutants, as well as for additional broader analysis of gametophyte functions.

## Conclusions

The gametophyte transcriptomes, particularly that of the male gametophyte, are distinct from those of sporophytic tissues, in agreement with results in *Arabidopsis* [12-15]. Analysis of RNA-seq data is useful for identifying previously unrecognized genes with gametophyte expression, particularly for the less accessible female. The male and female gametophyte transcriptomes are quite distinct from one another in the specific content of expressed genes, but some similarities in trends can be detected. Both gametophytes are more likely to express transposons/repetitive DNA than the sporophytic tissues examined, a phenomenon that has been reported previously in the pollen grain in maize [23] and *Arabidopsis* [36]. Male and female gametophytes are also both enriched compared with sporophyte tissues for expression of MADS box TFs and small DEFL signaling peptides. Whether these shared patterns reflect conserved haploid generation functions or convergence of function is unclear. Reduced mutation frequency in gametophyte-expressed genes also confirms the utility of these expression-based gene sets in identifying genes that are critical for gametophyte function and/or development. Comparison of retention rates for duplicate genes expressed in the pollen grain versus other tissues suggests that pollen function and competitiveness are more sensitive to gene balance, affecting evolution of gene pairs after genome duplication events.

## Materials and methods

### Sample preparation and RNA isolation

Plants for RNA were grown under long day conditions in the greenhouse in Stanford, CA or in summer field conditions in Corvallis, OR. Samples were collected between 11:00 and 11:30 am. Fresh mature pollen was collected upon shedding. For mature female gametophytes, ovules were dissected from ears and subjected to cell wall digesting enzymes to facilitate isolation of embryo sac tissue. ES and Ov samples were paired (that is, the ovule samples were produced from the tissue left over from embryo sac isolation). Three replicate RNA samples of each type were used to prime cDNA synthesis and amplification, with a slightly modified protocol for embryo sac and ovule tissue, due to the limited amount of starting material. We constructed libraries and produced paired-end and single-end sequence reads on the Illumina or SOLiD platform.

For isolation of ovule and embryo sac tissue whole ears were processed in the lab under a dissecting microscope. Ovules were isolated from ear florets with a silk length of approximately 10 cm by removing the silk and ovary wall with forceps and cutting the ovule at its base from the floret. Each ovule was immediately placed in a petri dish in a cell wall enzyme digesting mix of 0.75% pectinase, 0.25% pectolyase, 0.5% cellulase, 0.5% hemicellulase buffered in 0.55 M mannitol pH 5.0 for one hour after collecting the last ovule at 24 ± 1.0°C before embryo sac isolation according to Kranz *et al*. [81] and Yang *et al*. [26]. Embryo sacs (with some attached nucellus cells) were mechanically extracted from ovules using dissecting needles. The embryo sac samples and remaining ovule tissue (now lacking an embryo sac) were placed in separate microfuge tubes containing 500 μl of 0.55 M mannitol pH 5.0 until 15 to 20 embryo sacs and ovules lacking embryo sacs had been collected, and then samples were spun at 3,000 rpm for 1 minute and excess mannitol removed. Samples were homogenized in 400 μl of Trizol (Life Technologies, Grand Island, New York, USA) on a MixerMill300 (QIAGEN, Hilden, North Rhine-Westphalia, Germany) with a tungsten-carbide bead (QIAGEN) at high speed for 3 minutes, and RNA extracted according to manufacturer’s specifications to isolate total RNA. Mature, freshly shed pollen was collected from field-grown B73 plants, frozen in liquid nitrogen, and RNA extracted with Trizol (Invitrogen) and purified from the aqueous phase using RNEasy MinElute columns (QIAGEN). For whole seedling samples, all shoot tissue above the first leaf node was collected from 9-day-old B73 plants on the same day in liquid nitrogen, and RNA was isolated as for mature pollen.

cDNA libraries were generated from 0.5 to 20 μg total RNA. First strand cDNA was synthesized using the SMART PCR cDNA Synthesis Kit with SMART MMLV Reverse Transcriptase (Clontech Laboratories, Inc., Mountain View, California, USA) for pollen and seedlings or the SMARTer PCR cDNA Synthesis Kit with SMARTScribe Reverse Transcriptase (Clontech Laboratories, Inc.) for embryo sacs and ovules. The second strand was synthesized with the Advantage 2 PCR kit (Clontech Laboratories, Inc.). After second strand synthesis, cDNAs from the seedling and pollen samples (15 to 17 cycles), and the ovule and embryo sac samples (26 cycles), were amplified using the Advantage 2 PCR kit (Clontech) to produce sufficient cDNA for generating Illumina libraries. To identify ES/Ov sample pairs that had no contaminating post-fertilization (endosperm) tissue in any samples and no contaminating embryo sac tissue in the Ov samples, the resultant amplified cDNA was tested for presence and/or absence of several test genes for both B73 and W23 samples. PCR was performed with primers for the *Embryo Sac1* (*ES1*) (an embryo sac-specific gene) [28], *Embryo surrounding region1* (*ESR1*) (an endosperm-specific gene) [82], *EBE2* (a central cell and endosperm-specific gene) [83], *ubiquitin* (a constitutive gene), and *knox6* (a constitutive gene) [84]. Samples with no detectable *ESR1* transcripts in the ES or Ov samples or detectable *ES1* or *EBE2* transcripts in the Ov samples were used for sequencing. The cDNA libraries from B73 inbred samples were prepared for Illumina sequencing using a nebulizer for fragmentation and the Illumina Paired-End Sequencing preparation kit per the manufacturer’s protocol (Illumina catalogue numbers PE-102-1001 and PE-102-1002). Illumina sequencing was performed at the Oregon State University Center for Genome Research and Biocomputing. cDNA of the W23 samples was then used to prepare libraries and sequenced using the ABI SOLiD platform by Seqwright DNA Technology Services (Houston, TX, USA). Following mapping of reads to the maize genome and generation of FPKM values for all maize genes, a final round of quality control of the ES samples was performed. Because of the potential for variability introduced by the amplification of cDNA before sequencing and by variation in the amount of nucellar tissue left attached to the embryo sacs, a set of high confidence embryo sac-specific genes selected from the literature were examined in all the ES samples to determine which were sufficiently robust for further analysis (Additional file 22). These high confidence embryo sac-specific genes have been confirmed as embryo sac-specific in the context of the ovule either by *in situ* hybridization or by transgenic reporter analysis.

### Sequence analysis

80-mer paired-end reads were processed using the Illumina Genome Analysis Pipeline, version 1.5.0. TopHat, version 1.0.13, was used to align the RNA-seq reads to the maize genome (version ZmB73_5a.59) following several preprocessing steps, which included primer trimming, quality control filtering and length sorting [85]. Prior to aligning reads to the maize genome, reads matching maize repetitive sequences were filtered using the list available from the maize TE database [39]. Reads were aligned in paired-end mode when both reads of a pair passed all preprocessing steps, otherwise reads were aligned as singles. Empirical transcripts (etranscripts) were assembled from aligned data using Cufflinks, version 0.8.1, and FPKM expression data were generated using TopHat and Cufflinks [86]. All reads were then loaded into the gbrowse genome browser and a novel gbrowse plugin, QuantDisplay, was used to visualize the data. The sequence data are available at the Sequence Read Archive at NCBI, accession number SRP006965.

Given that TE databases have improved since the initial RefGen annotations were generated, BLAST was used to identify additional TE-related gene models in the FGS, WGS, and empirical transcript models [87]. All transcript model sequences were BLASTed against three different maize repeat databases (the MIPS Repeat Database, an updated version of the MTEC Transposable Element database [88], and the UTE database of unique TE sequences [89]). The top BLAST hit (ranked by bit score) for each was used to define whether a particular transcript model included TE-related sequences using a previously validated threshold (minimum hit length 50 bp, minimum identity 85%, minimum bit score 50) [41]. Sequences in the empirical transcript set not recognized as TE-related by this set of parameters were further screened by the RepeatMasker tool [90,91], which also detects simple sequence repeats. Sequences with lengths greater than 20% repetitive, or with >240 Smith-Waterman match score, were also classified as TE/repeat-related. Empirically predicted transcripts that did not correspond to annotated gene models and also were not recognized as TE- or repeat-related were subsequently analyzed by the BLAST2GO tool, to assess their potential protein coding capacity, either via BLAST or via a scan of the InterPro collection of protein signature databases [92].

### Quantitative RT-PCR analysis

Two control primer pairs were chosen, one each for ubiquitin and actin transcripts, each of which would target cDNA from multiple genes of each class to minimize effects of tissue-specific isoforms. All primer pairs were designed using Primer Select of the DNASTAR software package (Madison, Wisconsin, USA). Primer pairs were selected based on the following criteria: (1) they had to amplify within the last two or three exons to avoid potential problems from truncated, non-full-length cDNA; (2) they had to span an intron to distinguish cDNA from genomic DNA amplicons; (3) they had to have amplicons less than 150 bp to increase efficiency; (4) and they had to have a Tm of 60.0 ± 1.5°C. Primer pairs were then tested for efficiency on a pool of cDNA from all 12 samples. Primer pairs were only selected for further analysis if they produced a single amplicon and had an efficiency between 1.8 and 2.0. This produced a set of 22 genes for verification by qRT-PCR (genes and primers in Additional file 23).

### Analysis of gene sets based on expression

For identifying tissue-expressed or tissue-specific lists, a five read minimum per replicate and 0.1 FPKM minimum average were used. For the pollen and seedling tissues the average of the three B73 replicates was used to calculate a tissue-specific expression level. For the ES and Ov samples a more complicated method was used to combine data from three B73 replicates and three W23 replicates for each tissue type. The rationale for combining the B73 and W23 ES lists was supported by the analysis of the high confidence embryo sac-specific gene list that showed some genes had more robust comparisons with the B73 samples and others with the W23 samples (Additional file 22). Fewer genes were detected above 0.1 FPKM (Additional file 2) in the W23 samples than in the analogous B73 samples (24% fewer in ES, 21% in Ov). This is due in large part to the approximately 6,000 above-threshold gene models in the B73 FGS (some above 1,000 FPKM) that are associated with no reads in any of the W23 replicates. Many of the expression differences among these genes are likely caused by polymorphisms between W23 and B73 (indels or SNPs) that prevent mapping of the reads to the appropriate gene model; these polymorphisms may also include complete absence of these genes from W23. Presence/absence variation between maize inbreds can involve several thousands of sequences [93]. For all genes that had reads in the W23 samples, the average of the six samples (three W23 and three B73) was used to produce an embryo sac or ovule expression value. However, it is possible because of polymorphisms between W23 and B73 that some genes from the W23 samples would erroneously be assigned a FPKM value of zero because the reads do not match the reference B73 genome. For genes that had reads for either B73 ES or B73 Ov samples but zero reads in any of the six W23 samples, the B73 average was used instead of the average of the W23 and B73 samples together. This adjustment in the average FPKM was done for both tissue types with W23 and B73 samples: the ES and Ov samples. Consequently, the gene expression set for ES and Ov samples consisted of a hybrid of genes with an average over all six replicates and genes with an average over three B73 samples.

To identify tissue-enriched genes, pairwise comparisons between tissue expression levels were made for each tissue combination to identify genes with a two-fold expression difference between tissues. Tissue-enriched genes for this study were defined as genes two-fold higher in one tissue than all three other tissues and above a threshold of 0.1 FPKM. They were identified by determining the overlap between the gene lists of the three independent comparisons (for example, W23-B73 ES to W23-B73 Ov plus W23-B73 ES to B73 seedling plus W23-B73 ES to B73 MP; Additional file 7). To determine if there were any common functions in the gametophytes distinct from functions in the sporophyte, we identified a common gametophyte-enriched gene set - genes that were two-fold higher (threshold of 0.1 FPKM) for both gametophytes versus both sporophyte samples. First we identified the genes two-fold higher in the ES versus the Ov samples and two-fold higher in the ES versus the seedling samples. We similarly identified the genes two-fold higher in the MP versus the Ov and two-fold higher in the MP versus the seedling. Then the genes in common between these two sets were identified as a potential core gametophyte-enriched gene set of 591 genes (dual gametophyte-enriched Additional file 7).

Overlapping and tissue-exclusive gene sets were identified using the Venny online tool [94], and proportional Venn diagrams were produced using the online tool from BioInfoRx [95]. GO terms over-represented in the full transcriptomes of each tissue type and in the sets of tissue-enriched genes were identified using the online Agrigo GO Analysis Toolkit and Database for Agricultural Community [43,96], using the Maize ssp V5a gene ID settings.

To identify TF gene families overrepresented in tissue-enriched gene lists, the fraction of each tissue-enriched (that is, two-fold higher than the other three tissues) gene list made up of each TF family was compared with the expected value in the gene list based on the fraction of the FGS made up each TF family (Additional file 11). Chi-square values were calculated for each comparison between the observed and the expected number of TF family members, and TF families with significant enrichment were confirmed using a Fisher's exact test for the families with fewer than 200 members. Only TF families with an expected number above four were assayed and families with a *P* < 0.05 were considered significantly different from background.

To identify small peptide genes present in the WGS gene set but not annotated as being in these families, the Working Gene Set Peptide database was queried using BLAST at MaizeSequence [97] starting with the published founding family members. TF family lists were taken from the Grass Transcription Factor Database [48,49]. For all phylogenetic analyses, alignments were made using the ClustalW algorithm in MegAlign (DNASTAR). Phylogenies were produced from these alignments using MrBayes v3.2.0 using default settings for amino acid analysis [98]. Each analysis was performed for 100,000 generations or until the standard deviation of the split frequencies dropped below 0.05. The CLE, EAL, and ZPR gene families were each run for 100,000 generations; the DEFL family was run for 1,000,000 generations; the MADS family was run for 3,300,000 generations; the NAC family was run for 750,000 generations; the AP2-EREB family was run for 900,000 generations; the MYBR family was run for 950,000 generations; and the WRKY family was run for 350,000 generations. Phylogenetic trees were drawn from the MrBayes files using FigTree v1.4.0 [99].

For comparison of insertion frequencies in the tissue-enriched gene sets (seedling, pollen and embryo sac), datasets with the insertion locations for each of the three TE populations assessed were obtained from MaizeGDB [100] and imported into a Filemaker Pro database also containing the B73 Refgen v2 WGS and FGS feature locations (for example, exons, introns, coding sequence (CDS)). Each insertion location was subsequently mapped relative to these features in the WGS, and categorized based on this location (for example, Promoter -500 to -1, CDS_Exons, CDS_Introns). The number of insertions in each category and the average sizes in base pairs for each category were then derived by cross-referencing the expression sets with this insertion database.

## Additional files

Additional file 1: Table S1. Working gene set FPKM for all replicates and average FPKM per tissue type. Additional file 2: Table S2. Filtered gene set FPKM for all replicates and average FPKM per tissue type. Additional file 3: Table S3. Predicted intergenic gene models. **(A)** Summaries of characteristics of intergenic gene models (XLOCs). **(B)** Expression and position of non-repeat-related intergenic gene models. **(C)** Expression and position of repeat-related intergenic gene models. **(D)** Detailed BLAST2GO output for non-repeat-related intergenic gene models. Additional file 4: Table S4. All FGS genes with expression greater than or equal to 0.1 FPKM for each tissue, sorted highest to lowest as extracted from Additional file 2. Additional file 5: Table S4. Genes with duplicates in subgenomes 1 and 2 and singleton genes with enrichment (two-fold) in one tissue type versus the other three. Additional file 6: Table S6. Genes above 0.1 FPKM that are enriched in each tissue (that is, two- fold higher in the test tissue(s) than the other tissues). Additional file 7: Table S7. GO terms overrepresented in the full transcriptome of each tissue above 0.1 FPKM. **(A)** GO terms overrepresented in the full B73 seedling transcriptome for genes with average FPKM above 0.1; total of 27,564 genes, agrigo performed on 1 March 2013. **(B)** GO terms overrepresented in full B73 pollen transcriptome for genes with average FPKM above 0.1; total of 14,591 genes, agrigo performed on 1 March 2013. **(C)** GO terms overrepresented in full B73/W23 combined embryo sac transcriptome for genes with average FPKM above 0.1; total of 28,489 genes, agrigo performed on 1 March 2013. **(D)** GO terms overrepresented in the full B73/W23 combined ovules without embryo sacs transcriptome for genes with average FPKM above 0.1; total of 26,338 genes, agrigo performed on 1 March 2013. Additional file 8: Figure S1. Comparison of GO terms overrepresented in the full transcriptome of all four tissue samples. Overrepresented GO terms from the full transcriptome of each tissue type in Additional file 7 were compared to identify which GO terms are unique to each tissue and which are shared between tissues. Additional file 9: Table S8. GO terms overrepresented in each list of tissue enriched genes. **(A)** GO terms overrepresented in genes with seedling enrichment (two-fold higher in seedling compared to all other tissue types) and expressed above 0.1 FPKM; total of 8,066 genes, agrigo performed on 26 February 2013. **(B)** GO terms overrepresented in genes with pollen enrichment (two-fold higher in pollen compared to all other tissue types) and expressed above 0.1 FPKM; total of 2,224 genes, agrigo performed on 26 February 2013. **(C)** GO terms overrepresented in genes with B73/W23 embryo sac enrichment (two-fold B73/W23 combined embryo sac compared to all other tissue types) and expressed above 0.1 FPKM; total of 5,011 genes, agrigo performed on 26 February 2013. **(D)** GO terms overrepresented in genes with combined B73/W23 ovules enrichment (two-fold in B73/W23 ovules without embryo sacs compared to all other tissue types) and expressed above 0.1 FPKM; total of 1,770 genes, agrigo performed on 26 February 2013. **(E)** GO terms overrepresented in genes with enrichment in both B73 pollen and B73/W23 combined embryo sacs (two-fold compared to both seedling and ovule without embryo sacs) and expressed above 0.1 FPKM; total of 591 genes, agrigo performed on 26 February 2013. Additional file 10: Table S8. Analysis of pollen-expressed subgenome 2 genes. Additional file 11: Table S10. Representation of transcription factor families within each tissue-enriched gene list. Additional file 12: Figure S2. Phylogeny and expression of maize and *Arabidopsis* MADS transcription factor genes. Gene names in blue are part of the MP-enriched gene set. Gene names in red are part of the ES-enriched gene set. Gene names in magenta are part of the dual gametophyte-enriched gene set. *Arabidopsis* genes expressed in the embryo sac and pollen are from published reports [13,15,17,27,51-55]. Indication of gametophyte expression is different for maize and *Arabidopsis*. For maize it is called as positive if the gene is two-fold higher versus the three other tissues while in *Arabidopsis* it is measured as detectable expression, often by use of transgenic reporters. The yellow bar indicates the MIKC* group. The blue bar indicates the type 1 class γ group. The aqua bar indicates the type 1 class β group with no maize genes. The purple bar indicates the MIKC group. The green bar indicates the type 1 class α group. Posterior probability values are given at node positions. *Arabidopsis* genes begin with At. Additional file 13: Figure S3. Phylogeny and expression of maize NAC transcription factor genes. Gene names in red are part of the ES-enriched gene set. Posterior probability values are given at node positions. Additional file 14: Figure S4. Phylogeny and expression of maize AP2-EREB transcription factor genes. Gene names in red are part of the ES-enriched gene set. Posterior probability values are given at node positions. Additional file 15: Figure S5. Phylogeny and expression of maize MYBR transcription factor genes. Gene names in red are part of the ES-enriched gene set. Posterior probability values are given at node positions. Additional file 16: Figure S6. Phylogeny and expression of maize WRKY transcription factor genes. Gene names in red are part of the ES-enriched gene set. Posterior probability values are given at node positions. Additional file 17: Figure S7. Phylogeny and expression of maize CLE genes. Gene names in blue are part of the MP-enriched gene set. Gene names in red are part of the ES-enriched gene set. Gene names in magenta are part of the dual gametophyte-enriched gene set. Expression levels are indicated by color of the letter of each sample type with red meaning >10 FPKM, orange between 1 and 10 FPKM, green between 0.1 and 1 FPKM, blue greater than zero but less than 0.1 FPKM, and black having 0 reads. E, embryo sac expression; O, ovule without embryo sac expression; S, seedling expression; P, mature pollen expression. CLV3 of *Arabidopsis* is included for reference. Posterior probability values are given at node positions. Additional file 18: Figure S8. Phylogeny and expression of maize ZPR genes. Gene names in blue are part of the MP-enriched gene set. Gene names in red are part of the ES-enriched gene set. Gene names in magenta are part of the dual gametophyte-enriched gene set. Expression levels are indicated by color of the letter of each sample type with red meaning >10 FPKM, orange between 1 and 10 FPKM, green between 0.1 and 1 FPKM, blue greater than zero but less than 0.1 FPKM, and black having 0 reads. E, embryo sac expression; O, ovule without embryo sac expression; S, seedling expression; P, mature pollen expression. Both *Arabidopsis* (At) and rice (Os) genes are included for reference. Posterior probability values are given at node positions. Additional file 19: Table S11. Baseline frequency of gene models in the whole genome with at least one transposon insertion mapping to a particular region of the gene model. Additional file 20: Table S12. Frequency of tissue-enriched gene models with at least one transposon insertion mapping to a particular region of the gene model. Additional file 21: Table S13. Transmission data for *Ds* insertions in genes with and without enrichment in a gametophyte tissue. Additional file 22: Table S14. Verifying RNA-seq quality of embryo sac samples using high confidence embryo sac-specific genes. Additional file 23: Table S15. Primers for RT-PCR of test genes.

## Abbreviations

bp: base pair
ES: embryo sac
FGS: filtered gene set
FPKM: fragments per kilobase per million reads
GO: Gene Ontology
lncRNA: long noncoding RNA
MP: mature pollen
Ov: ovule with the embryo sac removed
qRT-PCR: quantitative reverse transcription polymerase chain reaction
RGS: rejected gene set
S: seedling shoot
SNP: single-nucleotide polymorphism
TE: transposable element
TF: transcription factor
WGS: working gene set
