# Supplementary figures and images for: Discovery of novel transcripts and gametophytic functions via RNA-seq analysis of maize gametophytic transcriptomes
Source: Genome Biol. 2014 Jul 31;15(7):414. doi: 10.1186/s13059-014-0414-2 (PMC4309534; doi:10.1186/s13059-014-0414-2)

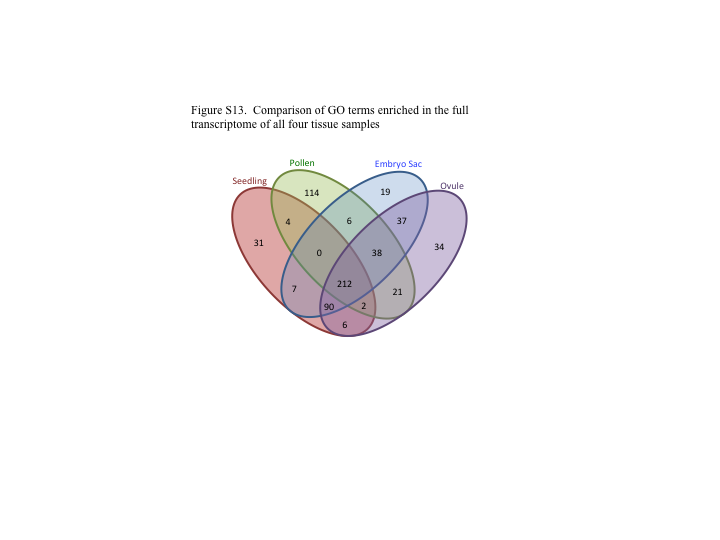

Supplement: Additional file 8: Figure S1. — Comparison of GO terms overrepresented in the full transcriptome of all four tissue samples. Overrepresented GO terms from the full transcriptome of each tissue type in Additional file 7 were compared to identify which GO terms are unique to each tissue and which are shared between tissues. [file 13059_2014_414_MOESM8_ESM.png]

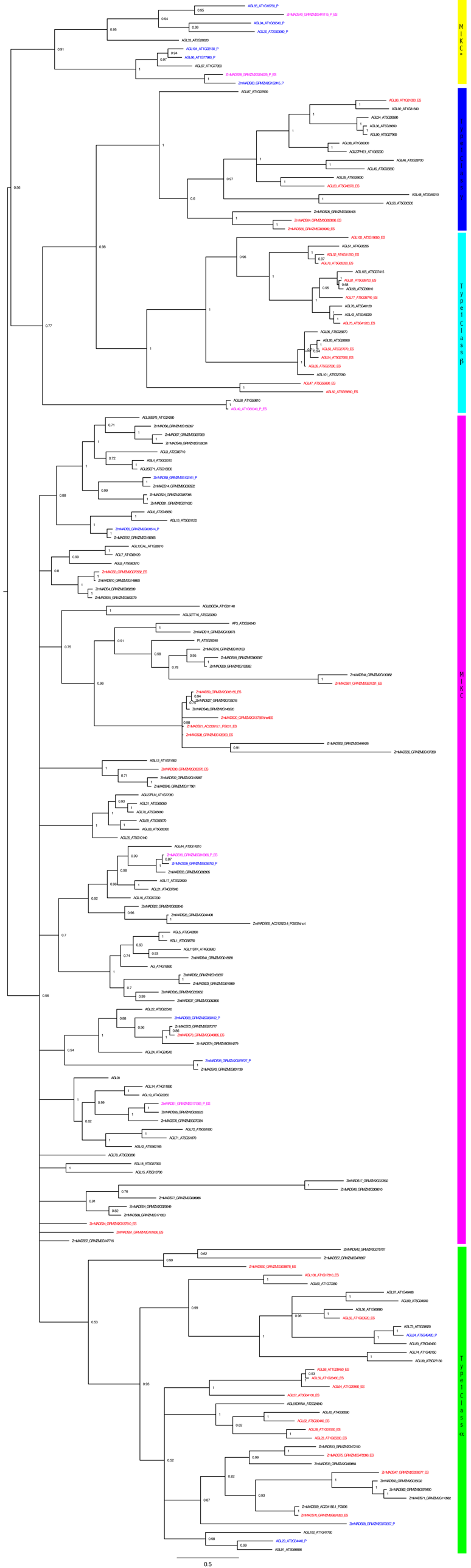

Supplement: Additional file 12: Figure S2. — Phylogeny and expression of maize and Arabidopsis MADS transcription factor genes. Gene names in blue are part of the MP-enriched gene set. Gene names in red are part of the ES-enriched gene set. Gene names in magenta are part of the dual gametophyte-enriched gene set. Arabidopsis genes expressed in the embryo sac and pollen are from published reports [13,15,17,27,51-55]. Indication of gametophyte expression is different for maize and Arabidopsis. For maize it is called as positive if the gene is two-fold higher versus the three other tissues while in Arabidopsis it is measured as detectable expression, often by use of transgenic reporters. The yellow bar indicates the MIKC* group. The blue bar indicates the type 1 class γ group. The aqua bar indicates the type 1 class β group with no maize genes. The purple bar indicates the MIKC group. The green bar indicates the type 1 class α group. Posterior probability values are given at node positions. Arabidopsis genes begin with At. [file 13059_2014_414_MOESM12_ESM.png]

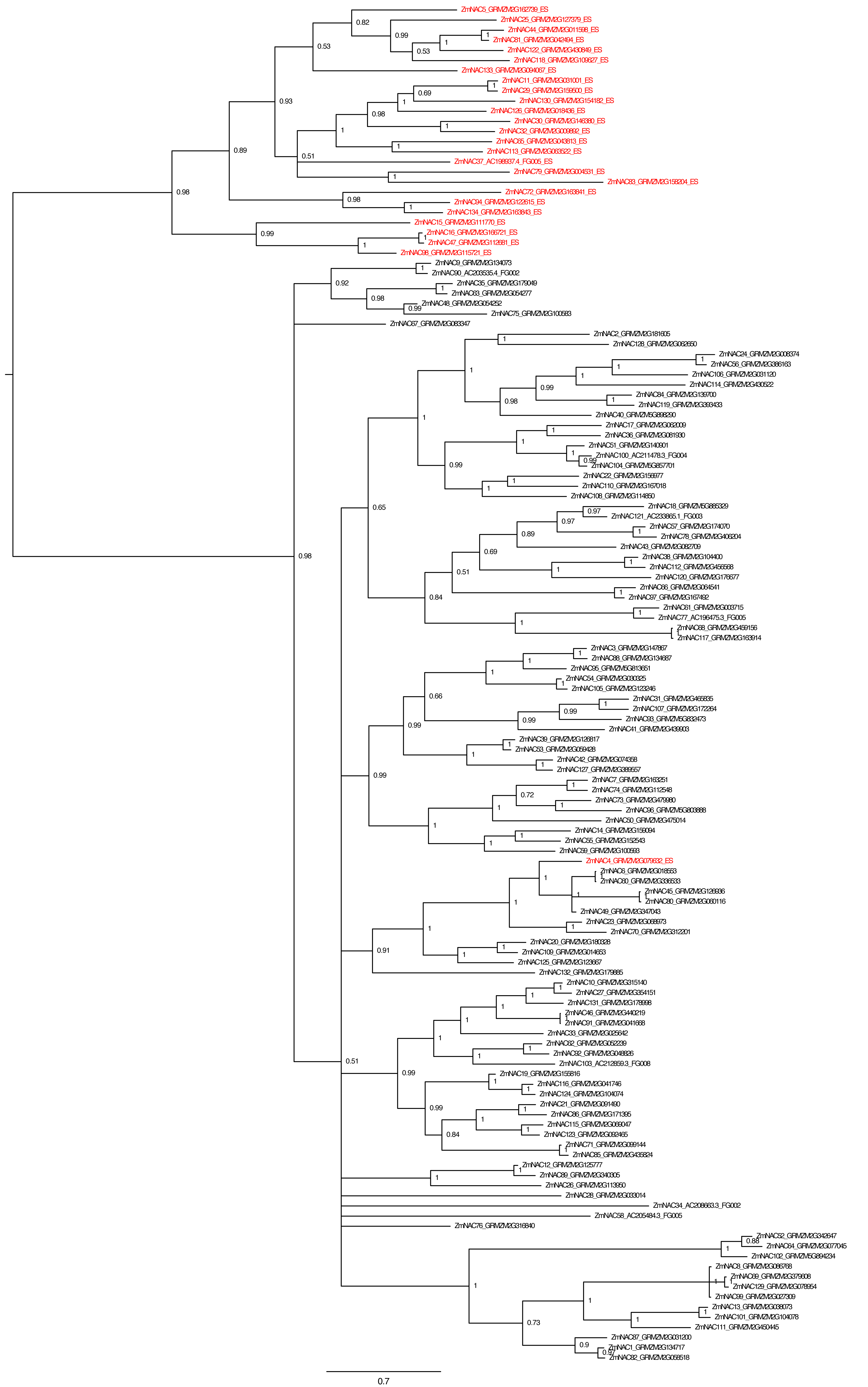

Supplement: Additional file 13: Figure S3. — Phylogeny and expression of maize NAC transcription factor genes. Gene names in red are part of the ES-enriched gene set. Posterior probability values are given at node positions. [file 13059_2014_414_MOESM13_ESM.png]

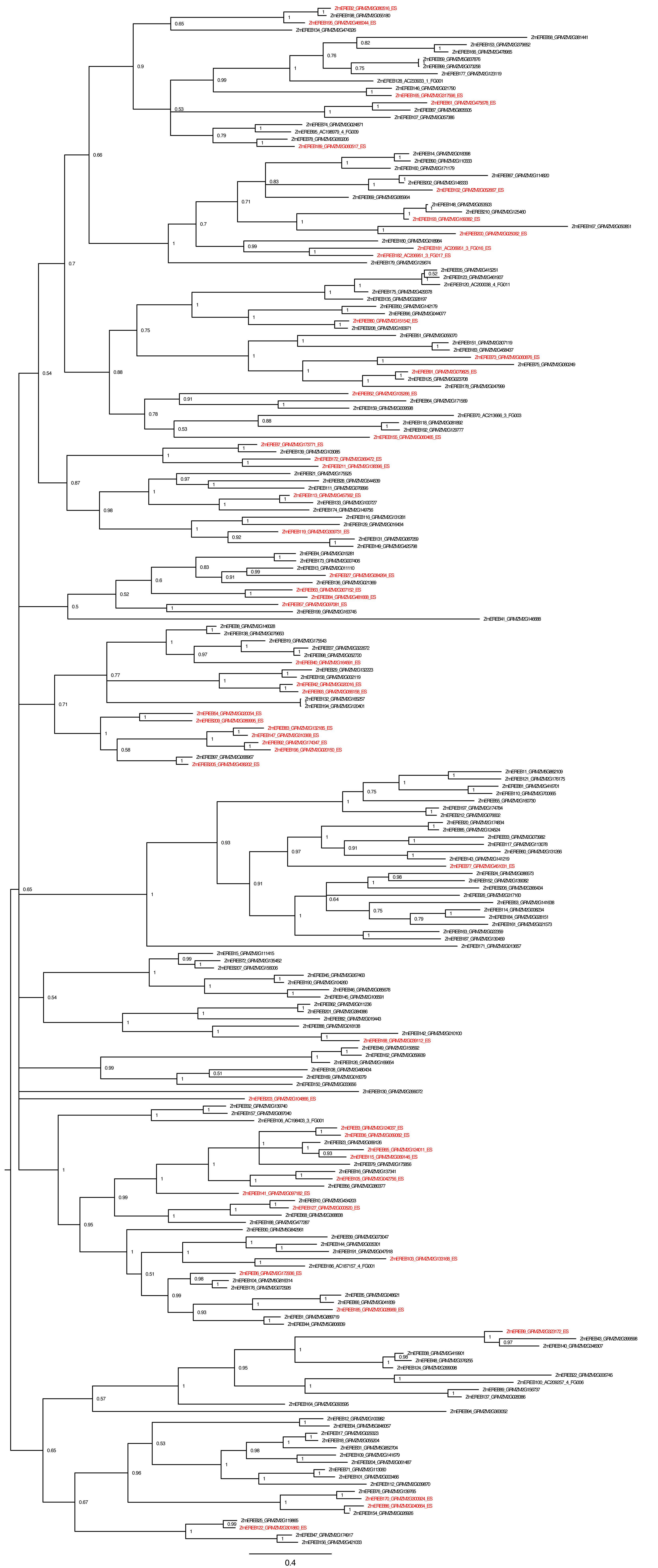

Supplement: Additional file 14: Figure S4. — Phylogeny and expression of maize AP2-EREB transcription factor genes. Gene names in red are part of the ES-enriched gene set. Posterior probability values are given at node positions. [file 13059_2014_414_MOESM14_ESM.png]

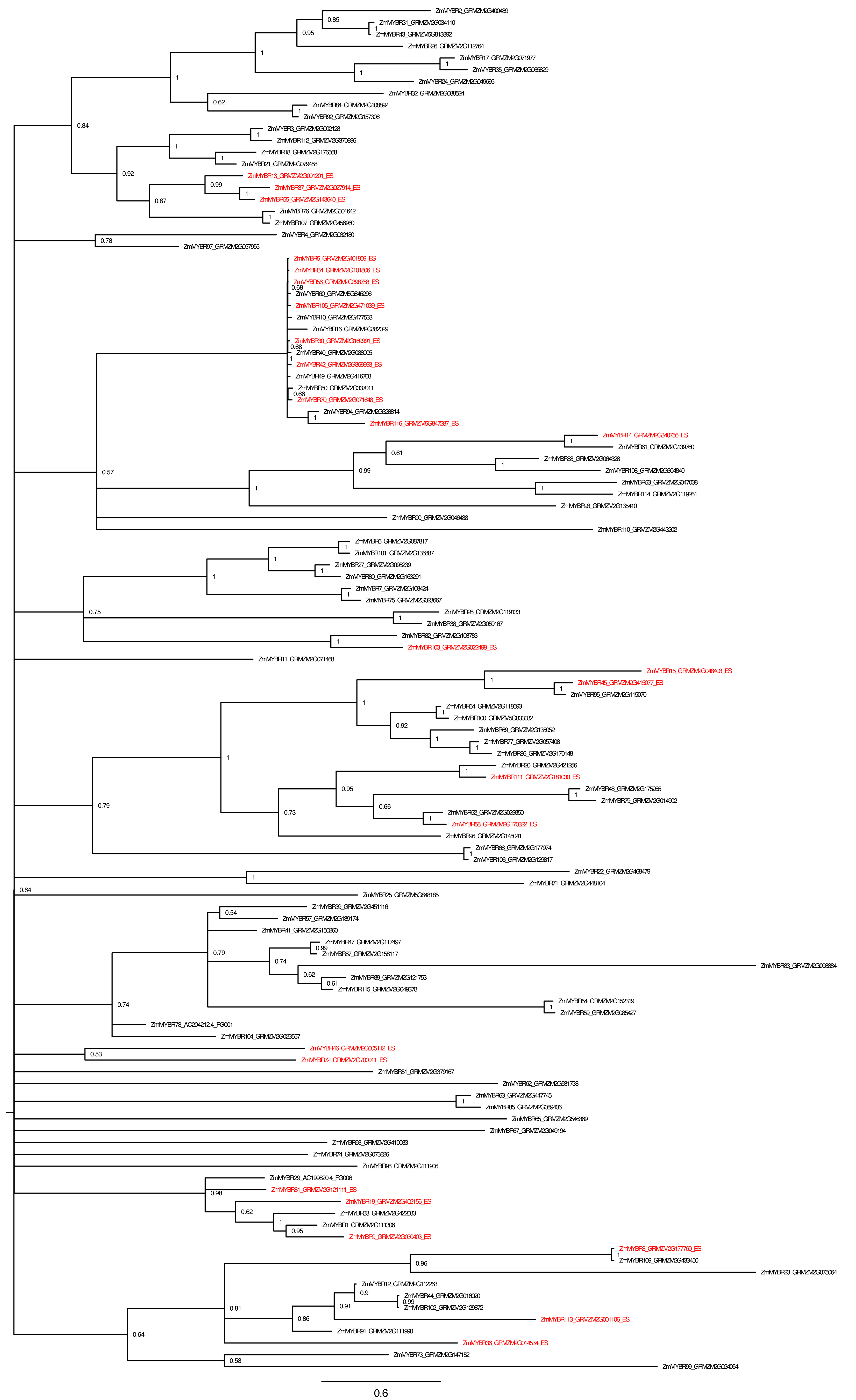

Supplement: Additional file 15: Figure S5. — Phylogeny and expression of maize MYBR transcription factor genes. Gene names in red are part of the ES-enriched gene set. Posterior probability values are given at node positions. [file 13059_2014_414_MOESM15_ESM.png]

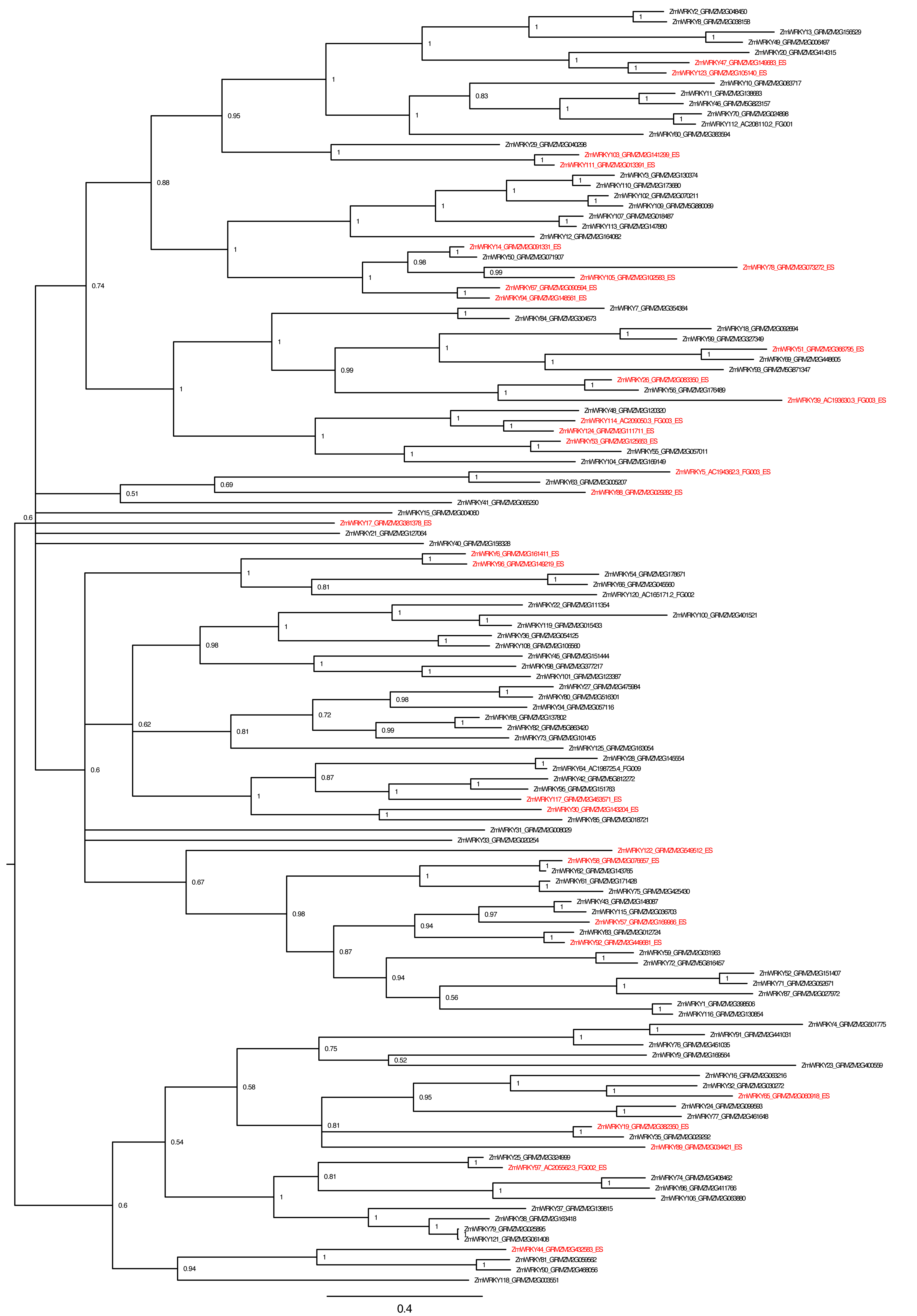

Supplement: Additional file 16: Figure S6. — Phylogeny and expression of maize WRKY transcription factor genes. Gene names in red are part of the ES-enriched gene set. Posterior probability values are given at node positions. [file 13059_2014_414_MOESM16_ESM.png]

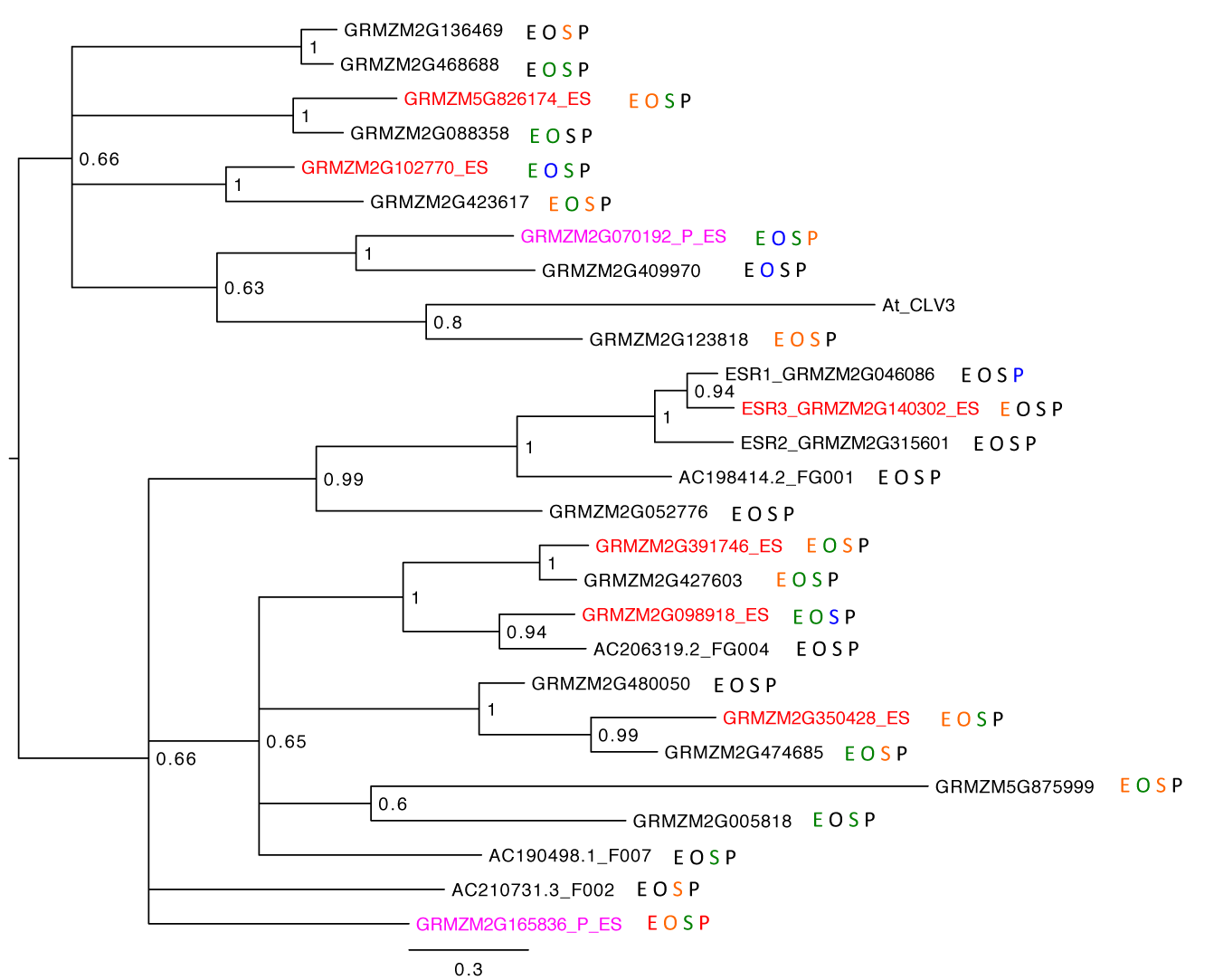

Supplement: Additional file 17: Figure S7. — Phylogeny and expression of maize CLE genes. Gene names in blue are part of the MP-enriched gene set. Gene names in red are part of the ES-enriched gene set. Gene names in magenta are part of the dual gametophyte-enriched gene set. Expression levels are indicated by color of the letter of each sample type with red meaning >10 FPKM, orange between 1 and 10 FPKM, green between 0.1 and 1 FPKM, blue greater than zero but less than 0.1 FPKM, and black having 0 reads. E, embryo sac expression; O, ovule without embryo sac expression; S, seedling expression; P, mature pollen expression. CLV3 of Arabidopsis is included for reference. Posterior probability values are given at node positions. [file 13059_2014_414_MOESM17_ESM.png]

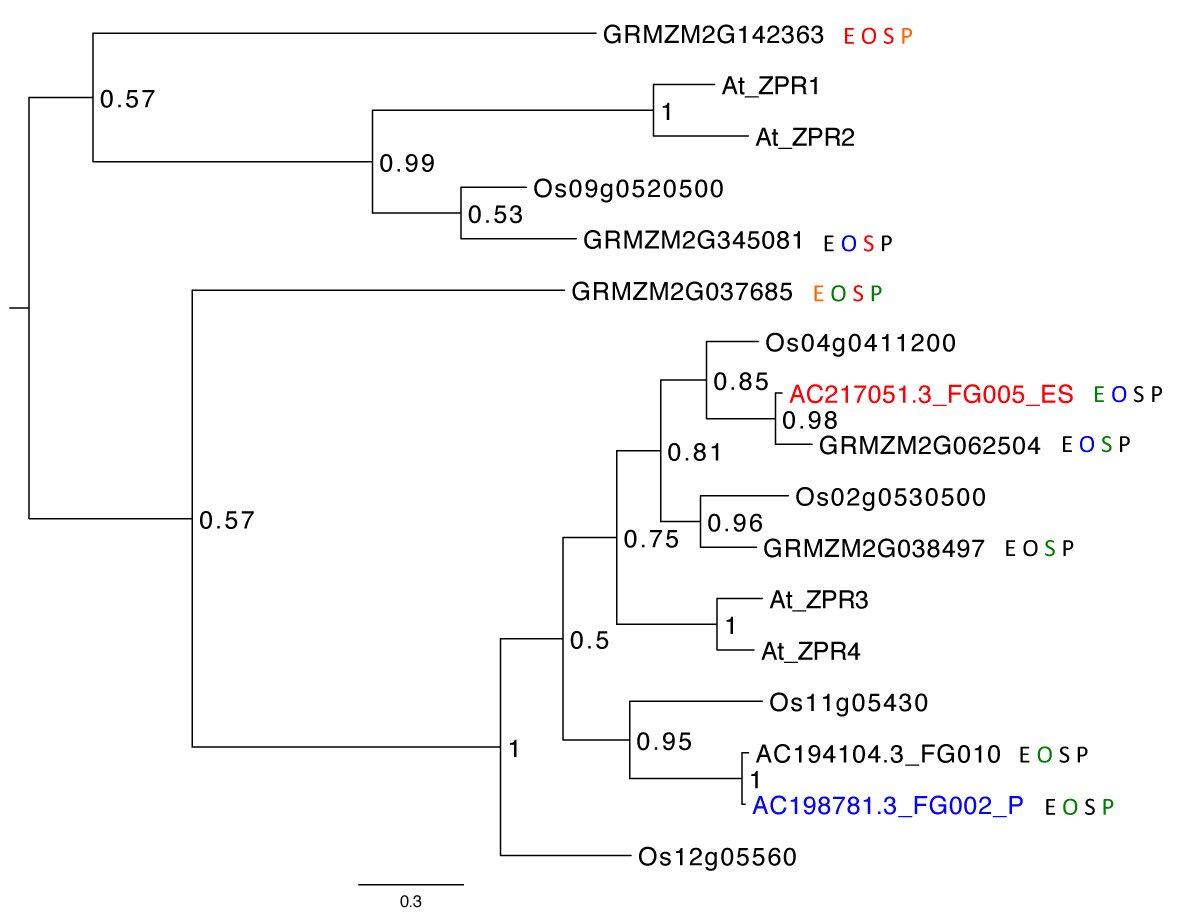

Supplement: Additional file 18: Figure S8. — Phylogeny and expression of maize ZPR genes. Gene names in blue are part of the MP-enriched gene set. Gene names in red are part of the ES-enriched gene set. Gene names in magenta are part of the dual gametophyte-enriched gene set. Expression levels are indicated by color of the letter of each sample type with red meaning >10 FPKM, orange between 1 and 10 FPKM, green between 0.1 and 1 FPKM, blue greater than zero but less than 0.1 FPKM, and black having 0 reads. E, embryo sac expression; O, ovule without embryo sac expression; S, seedling expression; P, mature pollen expression. Both Arabidopsis (At) and rice (Os) genes are included for reference. Posterior probability values are given at node positions. [file 13059_2014_414_MOESM18_ESM.png]
