# Supplementary material for: Discovery of novel transcripts and gametophytic functions via RNA-seq analysis of maize gametophytic transcriptomes
Source: Genome Biol. 2014 Jul 31;15(7):414. doi: 10.1186/s13059-014-0414-2 (PMC4309534; doi:10.1186/s13059-014-0414-2)
Supplement: Additional file 22: Table S14. — Verifying RNA-seq quality of embryo sac samples using high confidence embryo sac-specific genes. [file 13059_2014_414_MOESM22_ESM.docx]

| **Table S25. Verifying RNA-Seq Quality of Embryo Sac Samples Using High Confidence Embryo Sac Specific Genes** | | | | | | | | | | | | | | |
| --- | --- | --- | --- | --- | --- | --- | --- | --- | --- | --- | --- | --- | --- | --- |
|  | B73 inbred Illumina RNA-SEQ  (Working Gene Set) | | | | | | | W23 inbred SOLiD RNA-SEQ  (Filtered Gene Set) | | | | | | |
| GENE | Ov | ES | q | Sign | B73  ES1  RPM | B73  ES2  RPM | B73  ES3  RPM | Ov | Es | q | Sign | W23  ES1  RPM | W23  ES2  RPM | W23  ES3  RPM |
| EA1 (E&S) |  |  |  |  |  |  |  |  |  |  |  |  |  |  |
| GRMZM2G456746^a^ | 0.00 | 5.60 | 0.288 | no | 15.50 | 0.00 | 1.30 | 0 | 9.73 | 5.5E-07 | yes | 17.25 | 2.1 | 9.83 |
| GRMZM2G456746 |  |  |  | no |  |  |  |  |  |  |  |  |  |  |
| EBE2 (CC) |  |  |  |  |  |  |  |  |  |  |  |  |  |  |
| GRMZM2G167733^b^ | 0.78 | 2245.43 | 0.000 | yes | 6533.65 | 183.21 | 19.42 | 0.90 | 152.5 | 1.4E-09 | yes | 255.67 | 130.36 | 71.43 |
| GRMZM2G167733 |  |  |  | yes |  |  |  |  |  |  |  |  |  |  |
| EBE1 (CC) |  |  |  |  |  |  |  |  |  |  |  |  |  |  |
| GRMZM2G129157^b^ | 6.31 | 25.35 | 0.003 | yes | 61.99 | 9.53 | 4.54 | 0.16 | 10.37 | 2.4E-07 | yes | 19.13 | 2.27 | 9.7 |
| GRMZM2G129157 |  |  |  | yes |  |  |  |  |  |  |  |  |  |  |
| ES1 (E&S) |  |  |  |  |  |  |  |  |  |  |  |  |  |  |
| GRMZM2G012012^c^ | 0.00 | 29.50 | 0.287 | no | 86.20 | 0.05 | 2.24 | 1.01 | 3.1 | 0.60 | no | 5.58 | 0.00 | 3.55 |
| GRMZM2G012012 |  |  |  | no |  |  |  |  |  |  |  |  |  |  |
| ES3 (E&S) |  |  |  |  |  |  |  |  |  |  |  |  |  |  |
| GRMZM2G128301^c^ | 0.00 | 57.13 | 0.281 | no | 164.04 | 0.97 | 6.38 | 0.00 | 6.63 | 0.018 | yes | 12.63 | 1.21 | 6.05 |
| GRMZM2G128301 |  |  |  | yes |  |  |  |  |  |  |  |  |  |  |
| ES2 or ES4 (E&S) |  |  |  |  |  |  |  |  |  |  |  |  |  |  |
| GRMZM2G009359^c^ | 0.04 | 63.86 | 0.002 | yes | 182.72 | 2.41 | 6.45 | 0.00 | 7.68 | 6.9E-06 | yes | 7.94 | 13.8 | 7.68 |
| GRMZM2G009359 |  |  |  | yes |  |  |  |  |  |  |  |  |  |  |
| AE1 (CC) |  |  |  |  |  |  |  |  |  |  |  |  |  |  |
| GRMZM2G039942^d^ | 0.03 | 102.25 | 0.000 | yes | 268.61 | 7.23 | 30.91 | 0.00 | 43.27 | 2.3E-09 | yes | 56.85 | 30.62 | 42.34 |
| GRMZM2G039942 |  |  |  | yes |  |  |  |  |  |  |  |  |  |  |
| (CC&E) |  |  |  |  |  |  |  |  |  |  |  |  |  |  |
| GRMZM2G099353^d^ | 0.26 | 2513.88 | 0.000 | yes | 7117.92 | 16.73 | 406.98 | 0.51 | 58.21 | 5.0E-09 | yes | 84.46 | 32.85 | 57.32 |
| GRMZM2G099353 |  |  |  | yes |  |  |  |  |  |  |  |  |  |  |
| (S) |  |  |  |  |  |  |  |  |  |  |  |  |  |  |
| GRMZM2G006601^d^ | 0.33 | 3857.35 | 0.000 | yes | 11088.80 | 102.23 | 381.03 | 1.18 | 60.9 | 6.4E-07 | yes | 84.56 | 62.57 | 35.57 |
| GRMZM2G006601 |  |  |  | yes |  |  |  |  |  |  |  |  |  |  |
| (CC) |  |  |  |  |  |  |  |  |  |  |  |  |  |  |
| GRMZM2G481948^d^ | 0.02 | 72.90 | 0.000 | yes | 195.48 | 2.83 | 20.39 |  |  |  |  |  |  |  |
| GRMZM2G481948 |  |  |  | yes |  |  |  |  |  |  |  |  |  |  |
| (E&S) |  |  |  |  |  |  |  |  |  |  |  |  |  |  |
| GRMZM2G173023^d^ | 0.68 | 2568.76 | 0.000 | yes | 6680.96 | 265.78 | 759.53 | 3.81 | 424.28 | 5.1E-09 | yes | 680.1 | 408.75 | 184.01 |
| GRMZM2G173023 |  |  |  | yes |  |  |  |  |  |  |  |  |  |  |
| (CC) |  |  |  |  |  |  |  |  |  |  |  |  |  |  |
| GRMZM2G130320^e^ | 0.00 | 2.28 | 0.185 | no | 6.83 | 0.00 | 0.00 |  |  |  |  |  |  |  |
| GRMZM2G130320 |  |  |  | no |  |  |  |  |  |  |  |  |  |  |
| DSUL (E) |  |  |  |  |  |  |  |  |  |  |  |  |  |  |
| GRMZM2G006324^e^ | 0.22 | 0.27 | 0.53 | no | 0.65 | 0.00 | 0.00 | 0.00 | 5.30 | 7.9E-07 | yes | 11.3 | 0.49 | 4.1 |
| GRMZM2G006324 |  |  |  | no |  |  |  |  |  |  |  |  |  |  |
| (E) |  |  |  |  |  |  |  |  |  |  |  |  |  |  |
| GRMZM2G405804^d^ | 0.32 | 0.10 | -0.38 | no | 0.03 | 0.28 | 0.00 | 0.07 | 0.29 | 0.37 | no | 0.00 | 0.07 | 0.79 |
| GRMZM2G405804 |  |  |  | no |  |  |  |  |  |  |  |  |  |  |
| Blue=Comparison of all three B73 embryo sac samples to all three B73 ovule samples for Working Gene Set using Cufflinks  Green=Comparison of all three B73 Embryo Sac samples to all three B73 ovule samples using Gene Counter  Cell types of confirmed expression in parentheses above gene i.d. number next to gene name, if available: E=egg cell; S=synergid; CC=central cell.  ^a^ Marton, et al. (2005). Science. 307: 573-6.  ^b^ Magnard, et al. (2003). Plant Mol. Biol. 53, 821–836.  ^c^ Yang, et al. (2006). Planta. 224: 1004-1014  ^d^ Le, et al. (2005). Plant J 44, 167-178.  ^e^ Srilunchang, et al. (2010). Development 137:333–345 | | | | | | | | | | | | | | |

Called as significant

8 of 14 working gene set genes significantly enriched in B73 ES replicates using Cufflinks

9 of 14 working gene set genes significantly enriched in B73 ES replicates using GeneCounter

10 of 12 filtered gene set genes significantly enriched in W23 ES replicates using EdgeR
